# When the Going Gets Rough: The Significance of *Brucella* Lipopolysaccharide Phenotype in Host–Pathogen Interactions

**DOI:** 10.3389/fmicb.2021.713157

**Published:** 2021-07-15

**Authors:** Lauren W. Stranahan, Angela M. Arenas-Gamboa

**Affiliations:** Department of Veterinary Pathobiology, College of Veterinary Medicine and Biomedical Sciences, Texas A&M University, College Station, TX, United States

**Keywords:** *Brucella*, lipopolysaccharides (LPS), O-polysaccharide, vaccine, bacteria, Gram-negative, host–pathogen interaction, LPS

## Abstract

*Brucella* is a facultatively intracellular bacterial pathogen and the cause of worldwide zoonotic infections, infamous for its ability to evade the immune system and persist chronically within host cells. Despite the frequent association with attenuation in other Gram-negative bacteria, a rough lipopolysaccharide phenotype is retained by *Brucella canis* and *Brucella ovis*, which remain fully virulent in their natural canine and ovine hosts, respectively. While these natural rough strains lack the O-polysaccharide they, like their smooth counterparts, are able to evade and manipulate the host immune system by exhibiting low endotoxic activity, resisting destruction by complement and antimicrobial peptides, entering and trafficking within host cells along a similar pathway, and interfering with MHC-II antigen presentation. *B. canis* and *B. ovis* appear to have compensated for their roughness by alterations to their outer membrane, especially in regards to outer membrane proteins. *B. canis*, in particular, also shows evidence of being less proinflammatory *in vivo*, suggesting that the rough phenotype may be associated with an enhanced level of stealth that could allow these pathogens to persist for longer periods of time undetected. Nevertheless, much additional work is required to understand the correlates of immune protection against the natural rough *Brucella* spp., a critical step toward development of much-needed vaccines. This review will highlight the significance of rough lipopolysaccharide in the context of both natural disease and host–pathogen interactions with an emphasis on natural rough *Brucella* spp. and the implications for vaccine development.

## Introduction

Despite knowledge of its existence for over a century, brucellosis remains one of the most commonly reported zoonotic diseases worldwide (Pappas et al., 2006). A significant contributor to this is the fact that the causative Gram-negative bacterium, *Brucella*, is facultatively intracellular. This feature allows *Brucella* spp. to deviously persist within the host’s cells where it can evade many components of the immune system. On top of this, numerous animal species, particularly livestock, can carry the organism and readily transmit it to humans. Of the 12 identified species, the most frequently reported causes of human infection are *Brucella melitensis, Brucella abortus*, and *Brucella suis* with *B. canis* associated with fewer reported cases (Corbel, 1997; Pappas et al., 2008). Although cross-species infections are common with the first three strains, each shows a strong host preference with disease usually occurring in small ruminants, cattle, pigs, and dogs, respectively. *Brucella* spp. may also be classified according to their lipopolysaccharide (LPS) phenotype. The “classical” strains most commonly associated with human infection, *B. melitensis, B. abortus*, and *B. suis*, exhibit a smooth LPS while *B. canis* is naturally rough (Whatmore, 2009). *B. canis* owes this designation to the fact that its LPS conspicuously lacks the terminal O-polysaccharide (O-PS). Interestingly, roughness is not unique to *B. canis* as *B. ovis*, virulent in sheep, also lacks O-PS.

The rough phenotype of *B. canis* and *B. ovis*, defined by a lack of O-PS, is unusual as this trait is typically associated with attenuation in Gram-negative bacteria, yet both are fully virulent in their natural hosts (Moreno et al., 1984; Erridge et al., 2002). Like their smooth counterparts, both can cause reproductive disease in these species. In bitches, disease manifests as abortion while males commonly exhibit prostatitis and epididymitis (Carmichael and Kenney, 1968; Moore and Kakuk, 1969). Less commonly, dogs may present with diskospondylitis or uveitis (Kerwin et al., 1992). *B. ovis*, on the other hand, is a frequent cause of chronic epididymitis, orchitis, and infertility in rams with occasional induction of abortion in ewes (Buddle, 1956; Ficapal et al., 1998). One critical difference between these two strains, however, is that *B. canis* is zoonotic while *B. ovis* is not (Corbel, 1997).

The importance of developing new safe and effective vaccines against brucellosis cannot be overstated and this critical need is one aspect that applies to both smooth and rough *Brucella* spp. despite the difference in LPS phenotype. While canine brucellosis has historically been considered a pathogen predominantly of kenneled dogs, *B. canis* has been isolated with increasing frequency from stray dog populations as well as pets throughout the world (Hensel et al., 2018; Wang et al., 2018; Suárez-Esquivel et al., 2021). Although *B. canis* is perceived to be less virulent for humans, manifestation of human disease can occasionally be severe (Piampiano et al., 2000; Gul et al., 2009; Marzetti et al., 2013). Detection of *B. canis* in a kennel can also be devastating as the pathogen is highly contagious between dogs and those infected are frequently euthanized. *B. ovis*, although not zoonotic, can still result in significant economic losses for sheep farmers (Blasco, 1990). Unfortunately, no vaccine is currently available to protect against *B. canis* infection in dogs and the use of the commercially available Rev.1 vaccine, protective against *B. ovis* in sheep, is not approved for use in areas free of *B. melitensis*. Comprehending the components of the immune response required to protect against these naturally rough pathogens is crucial for development of new and effective vaccines.

This review will highlight the significance of rough LPS in the context of both natural disease and interaction with the immune response with an emphasis on natural rough *Brucella* spp. and the implications for vaccine development.

## Lipopolysaccharide Structure

**FIGURE 1:**
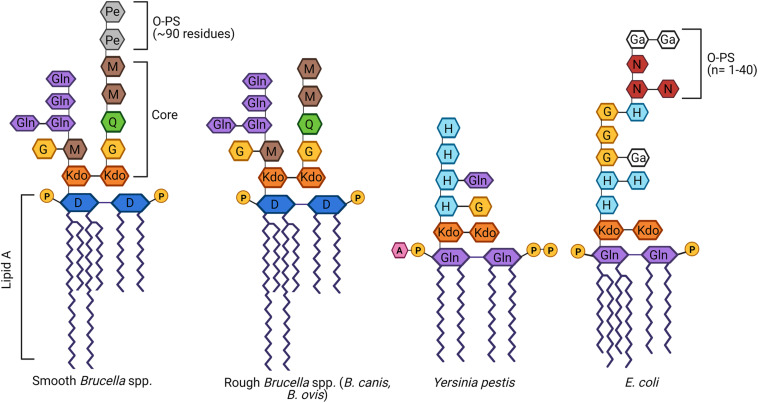
Schematic of the structure of lipopolysaccharide (LPS) of *Brucella* spp. in comparison to other Gram-negative bacteria. *Brucella* LPS is unusual in several ways, including the incorporation of very long chain fatty acids (VLCFAs) in the lipid A and a core oligosaccharide side branch composed of several glucosamine residues. In contrast, most other Gram-negative bacteria, best exemplified by *Escherichia coli*, possess a more compact lipid A and no core side branches. *Brucella* spp. may be categorized as smooth if they possess an intact O-polysaccharide (O-PS), as is the case with most zoonotic strains. Alternatively, *Brucella canis* and *Brucella ovis* are naturally rough, completely lacking the O-PS. While typically associated with attenuation, both rough *Brucella* spp. remain fully virulent in their natural hosts, as does the naturally rough *Yersinia pestis*. D, diaminoglucose; G, glucose; Ga, galactose; Gln, glucosamine; H, heptose; 3-deoxy-D-mannose-2-octulosonic acid, Kdo; M, mannose; N, *N*-acetylglucosamine; Pe, *N*-formylperosamine; Q, quinovosamine. Adapted from Mancilla (2015), Yang et al. (2019), and Singh et al. (2020). Created with Biorender.com.

As with most Gram-negative bacteria, *Brucella* spp. produce an LPS that plays a critical role in maintaining outer membrane integrity and survival within the host (Erridge et al., 2002; Mancilla, 2015). The classical smooth *Brucella* LPS is composed of three main components: (1) lipid A; (2) a polysaccharide core; and (3) O-PS composed of repeating glycosyl subunits (Smith, 2018; Figure 1). Lipid A is responsible for most of the endotoxic activity of LPS and its hydrophobicity allows it to anchor LPS to the outer membrane (Figure 1). Not only does lipid A play this key structural role, but it is the component of LPS that is recognized by Toll-like receptor (TLR)-4, one of the ways in which host immune cells can identify Gram-negative bacteria (Maldonado et al., 2016). In *Brucella*, lipid A is composed of a diaminoglucose backbone and is distinguished by a preponderance of C16 and C18 fatty acids as well as C28 and other very long chain fatty acids (VLCFAs), an unusual feature which results in a bulkier structure than most other Gram-negative bacteria (Iriarte et al., 2004; Mancilla, 2015).

The core oligosaccharide serves as a bridge between lipid A and O-PS and is important in maintaining the stability and rigidity of the outer membrane (Figure 1). In *Brucella* spp., the core is composed of two 3-deoxy-D-manno-2-octulosonic acid (Kdo) sugars, one of which is linked to the O-PS (Monreal et al., 2003). The other Kdo notably forms a unique branching side chain composed of glucosamine, glucose, and mannose (Gil-Ramírez et al., 2014; Fontana et al., 2016). As will be discussed in further detail below, the side branch appears to play an important role in survival within the host, helping to reduce immune recognition by TLR4 and enhancing resistance to complement and bactericidal peptides (Conde-Álvarez et al., 2012; Soler-Lloréns et al., 2014; Fontana et al., 2016). Although the structure of natural *B. canis* LPS has not been fully elucidated, the overall structure of the core oligosaccharide with side chain attached to a bulky lipid A embedded in the outer membrane is presumed to be similar if not the same between naturally smooth and rough *Brucella* spp.

The O-PS, a key virulence factor in smooth *Brucella* spp., remains the most studied component of this pathogen’s LPS (Allen et al., 1998; Jiménez de Bagüés et al., 2004; Mancilla, 2015; Figure 1). The lack of O-PS is what differentiates the LPS of *B. canis* and *B. ovis* from their smooth counterparts. In other Gram-negative bacteria, the O-PS typically exhibits a high degree of variability, allowing for strain differentiation by molecular methods (Wang et al., 2010; Smith, 2018). In contrast, *Brucella* O-PS is highly homogeneous, possibly due to its niche within the intracellular environment limiting horizontal transfer with other bacteria, as suggested by Mancilla (2015). The *Brucella* O-PS is composed of homopolymers of *N*-formylperosamine with minor variations depending on strain, in which “A-dominant” strains exhibit a linear α-1,2-linked polymer with approximately 2% α-1,3 linkages while the O-PS from “M-dominant” strains is a linear polymer of tetrasaccharide repeating units containing one α-1,3 linkage and three α-1,2-linked monosaccharide residues (Meikle et al., 1989; Kubler-Kielb and Vinogradov, 2013; Mancilla, 2015). Although not sufficient for speciation, serotyping using monoclonal antibodies directed against A or M epitopes can allow for distinction of different biovars within a particular smooth *Brucella* species (Alton et al., 1988). Further discussion of O-PS in this review will focus on the classical smooth *Brucella* spp. (*B. abortus, B. melitensis*, and *B. suis*). Although more recently identified *Brucella* species have been characterized as smooth (*B. neotomae, B. ceti, B. pinnipedialis, B. microti, B. papionis*, and *B. inopinata*), the complete structure of their LPS, including the O-PS, remains to be elucidated. Preliminary work has suggested that some of these strains not only lack typical O-PS epitopes but also suspected alterations to the core and/or lipid A, possibly accounting for a lack of zoonotic potential in the majority of these species (Cloeckaert et al., 1998; Baucheron et al., 2002; Zygmunt et al., 2012).

The mechanisms of *Brucella* O-PS and core oligosaccharide synthesis have been extensively reviewed (Cardoso et al., 2006; Mancilla, 2015) and the genes involved are summarized in Table 1. Interestingly, the lack of O-PS in *B. canis* and *B. ovis* appears to have evolved separately in an example of convergent evolution and the mutations resulting in their rough phenotypes are completely different (Vizcaíno et al., 2004; Zygmunt et al., 2009). *B. ovis*, for instance, lacks the entire genomic island-2 (GI-2) encompassing the key glycosyltransferases *wboA* and *wboB*, while *B. canis* retains this region (Vizcaíno et al., 2004; Tsolis et al., 2009).

**TABLE 1:** Genes involved in the synthesis of the core oligosaccharide and O-polysaccharide in *Brucella* spp.

LPS component	Gene(s)	Function	Comments	References
O-PS	*wboA*	Glycosyltransferase, O-PS synthesis	Encoded on genomic island-2, absent in *B.* ovis; Disrupted in RB51	Rajashekara et al., 2008; Mancilla et al., 2010
	*wboB*	Glycosyltransferase, O-PS synthesis	Encoded on genomic island-2, absent in *B. ovis*	Rajashekara et al., 2008; Mancilla et al., 2010
	*wbkA, wbkE*	O-PS polymerization		Zygmunt et al., 2009; Mancilla, 2015
	*gmd, per*	Perosamine synthesis		Godfroid et al., 1998, 2000
	*wbkC*	*N*-formylation of perosamine residues		Mancilla, 2015
	*wbkD, wbkF*	Bactoprenol priming	Both disrupted in *B. canis wbkD* disrupted in RB51	Zygmunt et al., 2009; Bricker et al., 2020
	*wzm, wzt*	O-PS transport to outer membrane	*wzt* truncated in *B. ovis*	Vizcaíno et al., 2004; Wattam et al., 2009
Core	wa**	Glycosyltransferase		Moriyón et al., 2004
	*pgm*	Phosphoglucomutase		Ugalde et al., 2000
	*wadB, wadC, wadD*	Glycosyltransferase	Synthesis of the branching side chain	Gil-Ramírez et al., 2014; Salvador-Bescós et al., 2018

Interestingly, *B. ovis* exhibits additional genetic differences from the smooth strains not identified in *B. canis*, such as the *B. ovis* pathogenicity island 1 (BOPI-1) which includes 28 open reading frames (ORFs) absent in other *Brucella* species and is required for *B. ovis* pathogenesis (Tsolis et al., 2009). At least some of these genes, such as the ABC transporter system, appear to compensate for a lack of alternative nutrient import pathways in *B. ovis* caused by separate mutations (Silva et al., 2011, 2014). Whether additional compensation for lack of O-PS is provided by these genes remains to be determined. *B. canis* and *B. ovis* are also distinguished by a higher number of pseudogenes, with the greatest number reported in *B. ovis*, a sign indicative of genome degradation (Tsolis et al., 2009; Wattam et al., 2009). It has been suggested that the narrower host range of these natural rough *Brucella* spp. is related to this process (Tsolis et al., 2009; Martín-Martín et al., 2011). However, the possibility of additional gain-of-function mutations leading to enhanced tropism for particular organ systems in their natural hosts despite a lack of O-PS (i.e., male genital tract for *B. ovis*) cannot be excluded (Tsolis et al., 2009; Moreno, 2014).

The indication that loss of O-PS occurred on two separate occasions amongst *Brucella* spp. suggests that this provided an evolutionary adaptation to *B. canis* and *B. ovis* and/or these two strains were able to compensate for its loss by additional but separate changes. This is a mystery within the field of brucellosis that is not yet unraveled but insights can be gained by understanding the roles O-PS plays for smooth *Brucella* spp. and how the natural rough strains compare in this regard.

Although separate from the LPS, outer membrane proteins (Omps) are another structural feature of the *Brucella* outer membrane that deserves special mention. Extensively reviewed elsewhere, Omps have been shown to be critical to outer membrane stability and appear to be especially important for the natural rough *Brucella* spp. (Cloeckaert et al., 2002; Roop et al., 2021). As will be discussed in greater detail later, certain Omps have been shown to contribute to complement and antimicrobial peptide resistance, to be essential for internalization of *B. ovis* into cells, and to inhibit antigen presentation (Caro-Hernández et al., 2007; Barrionuevo et al., 2008; Martín-Martín et al., 2008). Interestingly, the pattern of Omp expression, including Omp25/Omp31, in natural rough strains appears to differ from one another and with that of the smooth strains, although there is also variation amongst the classical smooth strains (Vizcaíno et al., 2004; Martín-Martín et al., 2009). *B. abortus*, for instance, lacks Omp31 and Omp25b, while *B. melitensis* lacks Omp31b (Cloeckaert et al., 2002). Additionally, while *omp25* deletion mutants of *B. abortus* and *B. melitensis* are attenuated in mice, this reduction in virulence is markedly more pronounced in *B. ovis*, although a separate study noted no attenuation in a *B. abortus* mutant (Edmonds et al., 2002; Manterola et al., 2007).

## The Biological Significance of LPS

**FIGURE 2:**
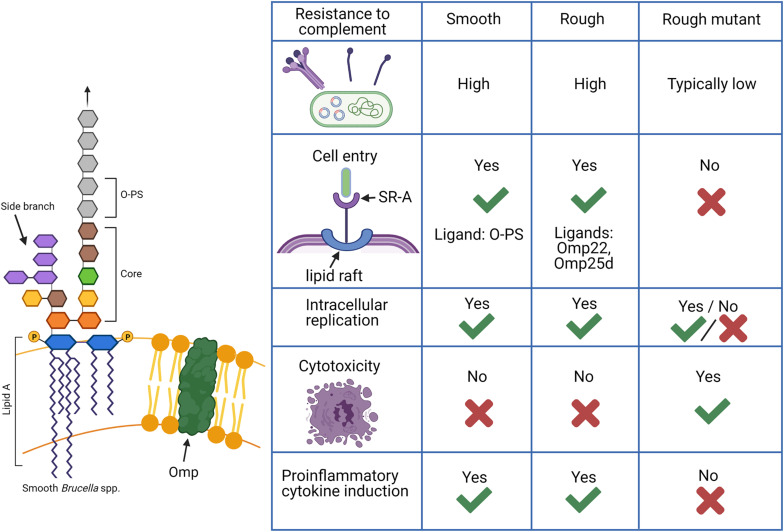
Functions of lipopolysaccharide (LPS) in *Brucella* spp. in comparison between smooth, rough, and rough mutant strains. Smooth *Brucella* spp. are known to be far more resistant to destruction by complement while rough mutants derived from these strains, in which the O-polysaccharide (O-PS) is not produced, are generally susceptible. Natural rough *Brucella* spp., such as *B. canis*, exhibit complement resistance as high or higher than their smooth counterparts despite a lack of O-PS with additional components such as the core oligosaccharide side branch and outer membrane proteins (Omps) making contributions. An additional difference is the ability of smooth and natural rough *Brucella* spp. to enter cells using lipid rafts and replicate intracellularly while rough mutants are typically unable to do this. Smooth *Brucella* spp. use O-PS as a ligand for the scavenger receptor, SR-A on host cells while natural rough *Brucella* spp. are thought to use outer membrane proteins for this interaction. Finally, rough mutants are known to cause cytotoxicity following cell infection and induce significant proinflammatory cytokine production while natural smooth and rough *Brucella* spp. do not. Such changes indicate that natural rough *Brucella* spp. are able to compensate for the loss of O-PS during infection. Created with Biorender.com.

The O-PS of the classical smooth *Brucella* spp. is a well-characterized virulence factor (Lapaque et al., 2005; Smith, 2018). The following sections will refer to rough mutants which are derived from smooth parent strains by deletion of any number of genes involved in O-PS synthesis and should be distinguished from natural rough *B. canis* and *B. ovis*. The majority of studies assessing the function of O-PS focus on comparing smooth strains and their rough mutants. Thus, far more is known about the relevance of the rough phenotype in rough mutants rather than in the natural rough *Brucella* spp. Nevertheless, natural rough strains appear to more closely mimic their smooth counterparts than rough mutants in features and interactions typically associated with expression of O-PS, such as resistance to complement, cellular entry and trafficking, and lack of induction of cytotoxicity and proinflammatory cytokine production, as summarized in Figure 2.

### Low Endotoxic Activity

Compared to most other Gram-negative bacteria, the LPS of *Brucella* is well known for exhibiting remarkably low endotoxin activity, being several hundred times less toxic than *Escherichia coli* LPS (Goldstein et al., 1992; Dueñas et al., 2004; Tumurkhuu et al., 2006). The low endotoxic activity of *Brucella* LPS is related to the structure of the lipid A and core. Endotoxicity is generally associated with the acyl chain number and length of lipid A, which is recognized by host cell Toll-like receptor 4 (TLR4) and its co-receptor MD2 (Erridge et al., 2002). The preponderance of VLCFAs in *Brucella* lipid A results in a bulky molecule that binds poorly with MD2 (Lapaque et al., 2005). In recent years, an additional explanation for the poor endotoxic activity of *Brucella* LPS has been revealed following the discovery of the lateral core oligosaccharide side branch.

Mutation of *wadC*, *wadB*, and *wadD*, glycosyltransferases required for the branch’s synthesis, has demonstrated the important roles this structure plays (Conde-Álvarez et al., 2012; Kubler-Kielb and Vinogradov, 2013). The side chain imparts a positive charge onto the core oligosaccharide that shields the more internal negative charges of the inner core and lipid A, thereby preventing effective interaction with TLR4/MD2 on host macrophages and dendritic cells (Conde-Álvarez et al., 2012; Fontana et al., 2016). This is important to note as the side chain is independent of the O-PS and thus likely performs the same function for the natural rough strains. Evidence toward this is the fact that purified LPS from *B. canis* and *B. ovis* exhibits low endotoxin-specific *Limulus* activity comparable to LPS of *B. abortus* and significantly less than that of the classic endotoxic LPS of *E. coli* (Moreno et al., 1984). It also bears mentioning that what little proinflammatory activity is stimulated by *Brucella* spp. is mediated largely by interaction of outer membrane lipoproteins with TLR2 on host cells, and the majority of these proteins are conserved across rough and smooth *Brucella* spp. (Giambartolomei et al., 2004; Roop et al., 2021).

### Defense Against Complement and Antimicrobial Peptides

One of the key functions of *Brucella* spp. O-PS is to provide resistance to destruction by complement and antimicrobial peptides (Allen et al., 1998; Eisenschenk et al., 1999). Specifically, O-PS has been shown to block access of C1q to outer membrane targets, which results in the known increased susceptibility of rough mutants to complement attack (Moreno et al., 1981; Corbeil et al., 1988; Allen et al., 1998; Eisenschenk et al., 1999). It is also known that O-PS is important in defense against antimicrobial peptides, including lysozyme, as evidenced by the increased sensitivity of rough mutants to these compounds (Riley and Robertson, 1984; Martínez de Tejada et al., 1995; Freer et al., 1996). Interestingly and in contrast to rough mutants, *B. ovis* and *B. canis* exhibit similar or occasionally greater resistance to complement and antimicrobial peptides compared to their smooth counterparts despite their rough phenotype (Martínez de Tejada et al., 1995; Martín-Martín et al., 2011). In one study, *B. canis* was actually found to be the most resistant strain overall to non-immune serum, low pH, H_2_O_2_, and cationic peptides (Martín-Martín et al., 2011). This indicates that O-PS is not the only factor capable of mediating such peptide resistance in *Brucella*.

The core oligosaccharide is one such structure involved in complement and bactericidal peptide resistance in both smooth and rough *Brucella* spp. (Soler-Lloréns et al., 2014; Fontana et al., 2016; Salvador-Bescós et al., 2018). The role of the core may also help explain an early report that *B. ovis* exhibits greater sensitivity to cationic peptides than rough mutants of *B. abortus* (Freer et al., 1999). This study utilized *B. ovis* REO198, a strain which possesses a core oligosaccharide defect, unlike *B. ovis* PA which has an intact core and exhibits enhanced resistance to cationic peptides (Martín-Martín et al., 2011; Pérez-Etayo et al., 2018). Interestingly, an intact core is also critical for resistance to antimicrobial peptides in other species of Gram-negative bacteria, including *Burkholderia cenocepacia* (Loutet et al., 2006).

Yet another feature that contributes to complement resistance in *Brucella* spp. are outer membrane proteins (Omps). As previously mentioned, the pattern of Omp expression differs both between smooth and rough *Brucella* spp. and between *B. ovis* and *B. canis.* It has been suggested that the differences in Omp expression largely account for the enhanced resistance of natural rough strains against complement and antimicrobial peptides despite the lack of O-PS, in contrast to many rough mutants (Caro-Hernández et al., 2007; Roop et al., 2021). These differences may also account for the finding that despite both being naturally rough, *B. ovis* is highly susceptible to the detergents deoxycholate, Triton X-100, and CHAPS while *B. canis* exhibits comparable or enhanced resistance compared to smooth *B. melitensis* and *B. abortus* (Martín-Martín et al., 2011). These findings are important to note as *B. canis* and *B. ovis*, despite both being rough, exhibit notable differences in host preference and zoonotic capability.

### Intracellular Survival and Replication

**FIGURE 3:**
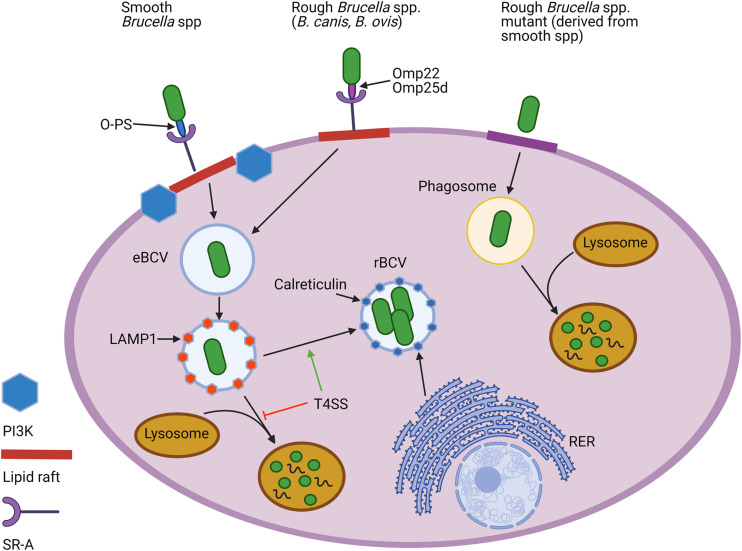
Cellular entry and intracellular trafficking of natural rough and smooth *Brucella* spp. and rough mutants derived from smooth strains. Both natural smooth and rough *Brucella* spp., but not rough mutants, enter macrophages using lipid rafts and utilize the scavenger receptor, SR-A, to do so. While the O-polysaccharide (O-PS) serves as the ligand for this interaction for smooth strains, rough *Brucella* spp. appear to rely on outer membrane proteins (Omps), including Omp22 and Omp25d. Lipid raft-mediated entry for smooth, but not natural rough *Brucella* spp., is reliant on the activity of phosphoinositide-3-kinase (PI3K). Once internalized, *Brucella* become engulfed in an endosomal *Brucella*-containing vacuole (eBCV) that alter associates with LAMP1. Expression of the type 4 secretion system (T4SS) allows some of the smooth and natural rough *Brucella* to avoid destruction by lysosomes and later associate with components of the rough endoplasmic reticulum (RER), such as calreticulin, to establish a replicative vacuole, or rBCV. In contrast, most rough mutants are unable to avoid lysosomal fusion and do not exhibit intracellular replication. Created with Biorender.com.

The importance of O-PS for smooth *Brucella* spp. in survival within their intracellular niche has been well established. O-PS can serve as an adhesin by binding to the scavenger receptor SR-A, allowing interaction with lipid rafts which facilitates entry of *Brucella* into the endocytic pathway (Porte et al., 2003; Kim et al., 2004) (Figure 3). From there, *Brucella* temporarily reside in an endosomal *Brucella*-containing vacuole (eBCV), in which the acidic pH signals the induction of genes encoding the Type-IV secretion system (T4SS). Following secretion of T4SS effectors, *Brucella* avoids destruction by lysosomes and traffics to the rough endoplasmic reticulum (RER), where it forms a replicative rBCV in which the bacteria may chronically persist (Celli, 2015). In contrast, rough mutants cannot enter cells via lipid rafts and vacuoles containing these bacteria fuse rapidly with lysosomes (Porte et al., 2003; Pei et al., 2008) (Figure 3). Rough mutants are also internalized more heavily and rapidly (Dornand et al., 2004; Tian et al., 2014). *B. ovis* and *B. canis*, however, more closely resemble the smooth strains in cellular entry and trafficking. For instance, both natural rough strains utilize lipid rafts to enter murine J774.A1 macrophages using the same SR-A receptor, although this interaction is conspicuously not dependent on phosphoinositide-3-kinase (PI3K) as it is with smooth strains (Martín-Martín et al., 2010) (Figure 3).

Some studies have noted that natural roughs penetrate HeLa cells and murine RAW 264.7 macrophages at a higher rate than smooths, although to a lesser degree than rough mutants (Freer et al., 1999; Sá et al., 2012). Other studies have noted that the binding and penetration of *B. ovis* occurs to a similar extent as smooth strains in murine macrophages and the same observation has been noted for *B. canis* in bovine neutrophils and human osteoblast cell lines (Delpino et al., 2009; Keleher and Skyberg, 2016). Given the lack of O-PS in both natural and mutant rough strains, it is unclear which molecule(s) may serve as a ligand for SR-A although it is possible that certain Omps may play this role. Two possibilities are Omp25d and Omp22 which are required for entry and replication in murine RAW 264.7 or J774.A1 macrophages by *B. ovis* but not by smooth strains in which O-PS predominantly determines cell entry (Manterola et al., 2007; Martín-Martín et al., 2008).

Following entry, rough mutants are rapidly destroyed via fusion with lysosomes and are typically unable to replicate intracellularly or exhibit reduced levels of replication within murine J774A.1 macrophages and human monocytes, depending on the mutation (Porte et al., 2003; Rittig et al., 2003; Jiménez de Bagüés et al., 2004) (Figure 3). Despite earlier reports showing the inability of *B. canis* to do this, it has since been shown that both *B. canis* and *B. ovis* are fully capable of intracellular replication in macrophages and epithelial cells, with replication of *B. canis* noted in HeLa cells, murine RAW 264.7 macrophages, and canine trophoblasts and that for *B. ovis* noted in Vero cells, murine J774.A1 macrophages, and HeLa cells (Detilleux et al., 1990; Martín-Martín et al., 2008; Eskra et al., 2012; Silva et al., 2014; Chacón-Díaz et al., 2015; Fernández et al., 2017). *B. ovis* can replicate in both canine and ovine macrophages and *B. canis* exhibits the same ability (Eckstein et al., 2020). The T4SS appears to be just as important for allowing intracellular replication in the natural roughs as in the smooths as mutants in *virB* genes or *vjbR* of *B. ovis* and *B. canis* exhibit significant attenuation and inability to replicate within ovine macrophages and mice or HeLa cells, murine RAW 264.7 macrophages, and canine DH82 dendritic cells, respectively (Sá et al., 2012; Chacón-Díaz et al., 2015; Macedo et al., 2015; Stranahan et al., 2020).

Interestingly, while phagosomes containing rough mutants fuse rapidly with lysosomes and many are unable to progress beyond this point to establish replication in the rBCV, *B. ovis* and *B. canis* appear to eventually be able to reach the replicative niche and replicate to levels identical to smooth strains in HeLa cells and murine RAW264.7 macrophages (Silva et al., 2014; Chacón-Díaz et al., 2015) (Figure 3). Silva et al., 2014 demonstrated that BCVs containing *B. ovis* in HeLa cells interact early in infection with lysosomes, as shown by association with LAMP1, and that this marker is later excluded with formation of the typical rBCV associated with calreticulin, a marker of the RER. Formation of myelin figures associated with vacuole-enclosed bacteria in ovine macrophages also suggests that *B. ovis* may continue in the pathway followed by smooth strains with formation of an autophagic vacuole for cellular egress (Macedo et al., 2015). Interestingly, *B. canis* also exhibits high levels of lysosome fusion in the first few hours of cellular infection, comparable to rough mutants and significantly less than smooth strains, but is able to replicate after 48 h comparable to *B. abortus* levels in HeLa cells and murine J774.A1 or RAW264.7 macrophages, (Porte et al., 2003; Chacón-Díaz et al., 2015). Nevertheless, differences have been observed: *B. ovis* shows later evasion of lysosome fusion compared to smooth strains, with this change happening after 48 h in ovine macrophages instead of the 24 h noted for *B. abortus* in murine J774.A1 macrophages (Starr et al., 2008; Silva et al., 2014). For *B. canis*, a similar trend has been noted in which colocalization of the bacterium with calnexin, another RER marker, does not occur in HeLa cells until 48 h post-infection (Chacón-Díaz et al., 2015). This delay in avoidance of phagosome-lysosome fusion may help explain why earlier studies, which did not extend time points beyond 24 h, demonstrated prominent and rapid phagosome fusion with lysosomes using natural rough strains in contrast to smooth strains, with the conclusion that natural roughs could not replicate intracellularly (Porte et al., 2003; Rittig et al., 2003). It has been well established that natural rough *B. ovis* and *B. canis* can replicate within cells both from their natural hosts as well as mice and humans.

### Prevention of Apoptosis and Cellular Activation

O-PS-mediated entry by smooth *Brucella* spp. via lipid rafts has been shown to inhibit caspase-2-mediated apoptosis in phagocytes, including murine J774.A1 and RAW264.7 macrophages and human macrophages (Fernandez-Prada et al., 2003; Pei et al., 2006; Chen and He, 2009). Rough mutants, on their other hand, are well known for induction of cytotoxicity which is dependent on the T4SS in the same cell types (Fernandez-Prada et al., 2003; Pei et al., 2008). While the mechanisms behind this are incompletely understood, it appears that overexpression of the T4SS occurs during cellular infection by rough mutants, leading to over-secretion of effector proteins which activate the IRE1α pathway of ER stress (Pei et al., 2008; Li et al., 2017). Interestingly, this seems not to be the case with natural rough strains, where *B. canis* and *B. ovis* resemble their smooth counterparts and have been repeatedly shown not to induce cytotoxicity in HeLa cells, murine J774.A1 and RAW 264.7 macrophages, or human lung epithelial cells (Martín-Martín et al., 2008; Ferrero et al., 2009; Chacón-Díaz et al., 2015).

O-PS-mediated uptake of smooth *Brucella* spp. is also known to induce only low levels of proinflammatory cytokines in phagocytes, especially in comparison to other Gram-negative pathogens (Jiménez de Bagüés et al., 2004; Billard et al., 2007). This is an attribute which has been added to the list of ways in which *Brucella* spp. can subvert the host immune response. Nevertheless, it is important to note that despite this low induction of inflammatory cytokines, inflammation is still a classic feature of brucellosis and although typically milder compared to that caused by most other bacteria, the chronic stimulation of low-level inflammation by persisting *Brucella* spp. frequently results in tissue damage (Baldi and Giambartolomei, 2013). In contrast to smooth strains, penetration by rough mutants leads to significant production of proinflammatory cytokines in human monocytes or macrophages and murine J774.A1 macrophages (Fernandez-Prada et al., 2003; Rittig et al., 2003; Pei et al., 2008). *B. canis* and *B. ovis*, like the smooth strains, also induce minimal to no proinflammatory cytokine production in cells while rough mutants, including RB51, stimulate high levels of TNF-α and IL-12 in murine J774.A1 macrophages, although this pattern has yet to be investigated in macrophages derived from their natural hosts (Martín-Martín et al., 2010). A possible explanation for this is that, like the smooth strains, *B. canis* and *B. ovis* enter cells via lipid rafts and follow the same general intracellular trafficking pathway, albeit with some delay, while the contrasting entry by rough mutants triggers a strong cellular response. The differences in the intracellular pathway between natural rough and smooth strains, such as the ligand for SR-A and timing of lysosome evasion, have been proposed to account for the further reduced inflammatory response of *B. ovis* and *B. canis* when compared with smooth strains in mice and natural hosts (Galindo et al., 2009; Chacón-Díaz et al., 2015; Roop et al., 2021).

While it is apparent that O-PS serves directly or indirectly as a key virulence factor for smooth *Brucella* spp., the lack of this molecule in natural rough strains appears to be compensated for, at least partially, by alterations in Omp expression and the continued presence of the lateral core oligosaccharide side branch (Mancilla, 2015; Roop et al., 2021). The possibility that a rough phenotype in itself could serve an additional advantage to *Brucella* spp. is intriguing and this idea can best be contemplated after understanding the reasons why other Gram-negative pathogens, such as *Pseudomonas aeruginosa* and *Yersinia pestis*, have either temporarily or permanently lost their O-PS.

## The Advantages of Going Rough

Naturally smooth *Brucella* spp. can undergo temporary or permanent loss of O-PS under both laboratory conditions and during the course of infection within their natural hosts (Mancilla, 2015). This process is exemplified by RB51, a spontaneous and stably rough vaccine strain that arose after repeated passage on antibiotic-containing media and has been widely utilized in studies investigating the function of *Brucella* O-PS (Schurig et al., 1991). Another example is the now out of use *B. abortus* 45/20 vaccine strain, which acquired a rough phenotype after repeated passage through guinea pigs (Moriyón et al., 2004).

Dissociation from a smooth to a rough phenotype and the potential advantages of this change for the bacterium have been studied for many years with various Gram-negative bacteria. Reeves (1995) suggested that the tendency of Gram-negative bacteria to become rough under laboratory conditions is due to the necessity of O-PS for survival only in the presence of the host immune response. Requiring energy to produce, there appears to be a selective advantage to loss of O-PS in situations where it is not needed, as in liquid culture (Maldonado et al., 2016). Isolation of rough variants of smooth Gram-negative bacteria in samples from infected hosts, including *Brucella*, also suggests that there may be an adaptive advantage to loss of O-PS in evading the host immune system, particularly in chronic infections (Turse et al., 2011; Mancilla et al., 2012; Szabo et al., 2017). Although this seems to contradict the statement that Gram-negative bacteria require O-PS to resist destruction by the host immune system, namely via complement, it appears that various bacteria will sacrifice the protection offered by a complete O-PS in exchange for additional survival benefits such as avoidance of a robust antibody response and improved access to preferred host-cell types (Maldonado et al., 2016). Could it be that a rough phenotype in *B. canis* or *B. ovis* is not only compensated for by changes to the outer membrane but may actually benefit these organisms by assisting in maintenance of a chronic infection? While the answer to this question remains uncertain, several other Gram-negative bacteria develop a rough phenotype during chronic infections.

One example is *P. aeruginosa*, particularly in patients with cystic fibrosis (CF). While isolates from acutely infected patients or the environment typically have a smooth phenotype, most from chronically infected patients with CF are rough (Hancock et al., 1983; Goldberg and Pler, 1996; Lam et al., 2011). The reasons behind this phenomenon and its propensity for occurring in CF patients are not fully understood, but it has been suggested that while rough *P. aeruginosa* strains are more serum-sensitive, the reduced immunostimulatory potential following loss of the O-PS may contribute to immune evasion and survival in chronic lung infections. Like their smooth counterparts, *B. canis* and *B. ovis* can avoid activation of macrophages along with subsequent proinflammatory cytokine production (Martín-Martín et al., 2010). *B. canis* in particular has been shown to be less inflammatory in the mouse model in terms of pathologic changes and cytokine production and induces less ROS production in infected humans than smooth strains despite similarly low levels of cytokine induction noted by the previously mentioned *in vitro* studies (Usta et al., 2012; Chacón-Díaz et al., 2015; Stranahan et al., 2019). As suggested by Chacón-Díaz et al. (2015), *B. canis* might therefore be an even stealthier pathogen in its natural host than smooth *Brucella* spp., although this possibility remains to be tested in dogs or canine cells. The rough phenotype of *B. canis* could possibly serve an advantage by reducing immune stimulation, allowing it to establish long-lasting, asymptomatic infections, while simultaneously avoiding the serum sensitivity observed with other rough Gram-negative bacteria via adjustments to the structure of its outer membrane.

Another example of a bacterium that benefits from loss of O-PS is *B. cenocepacia*, which also undergoes this change during chronic infection of CF patients (Evans et al., 1999). Assumption of a rough phenotype facilitates internalization into macrophages, a niche that this facultatively intracellular pathogen could subsequently exploit to favor persistence (Saldías et al., 2009; Schwab et al., 2014). Enhanced phagocytosis by macrophages is also observed in both rough mutants and, depending on the study, with the naturally rough *B. ovis* (Detilleux et al., 1990; Tian et al., 2014). Whether this enhanced entry could also assist rough *Brucella* spp. in establishing their preferred intracellular niche early in infection is unknown. It also bears mentioning that enhanced entry of natural and mutant rough *Brucella* spp. has been noted in various cell types beyond macrophages, including epithelial and endothelial cells and without associated replication (Ferrero et al., 2009, 2011).

The ability to exploit new host cell receptors by exhibiting a rough phenotype might serve a third possible advantage for Gram-negative bacteria. For instance, the normally smooth *Helicobacter pylori* is able to bind to a receptor called TFF1 on injured gastric mucosa specifically through rough LPS, as demonstrated by work examining the interaction of TFF1 with purified *H. pylori* rough LPS or a rough mutant (Reeves et al., 2008; Dolan et al., 2012). Both naturally rough and smooth *Brucella* spp. utilize the scavenger receptor, SR-A (CD36) to enter macrophages (Pei et al., 2008; Martín-Martín et al., 2010). Whether the exposure of the core and additional outer membrane components such as Omps could allow naturally rough *Brucella* spp. to exploit additional receptors unavailable to smooth strains in their natural hosts is uncertain. That this may be the case is supported by the finding that O-PS may interfere with interaction of Omps with anti-*Brucella* antibodies (Bowden et al., 1995).

Another Gram-negative bacterium deserves special mention here: *Y. pestis*. Like *B. canis* and *B. ovis*, *Y. pestis* has permanently lost its O-PS yet remains a virulent pathogen. Although the structure of its LPS differs significantly from that of *Brucella* (Figure 1), *Y. pestis* exhibits several similarities in its behavior such as the ability to survive in macrophage phagosomes and inhibition of fusion with lysosomes to allow for intracellular replication (Pujol et al., 2009; Connor et al., 2018). The rough LPS provides a selective advantage to this pathogen by enabling exposure of the protein Ail at the cell surface, resulting in thickening and rigidification of the LPS which actually promotes serum resistance (Singh et al., 2020). This is reminiscent of the high serum resistance of the natural rough *Brucella* but the exact structural modifications resulting in this enhanced resistance for *B. ovis* and *B. canis* have not been fully unraveled (Caro-Hernández et al., 2007; Martín-Martín et al., 2011). Additionally, the rough *Y. pestis* is able to use its exposed core to bind SIGNR1 (CD209b), a receptor on antigen-presenting cells, facilitating its dissemination to various organs (Yang et al., 2019). Interestingly, experimentally derived smooth *Y. pestis* actually has an impaired ability to cause systemic infection in mice, leading to the proposal that loss of O-PS was key to the evolution of *Y. pestis* as a highly virulent pathogen (Yang et al., 2019). As described above, the absence of O-PS in *B. canis* and *B. ovis* coincides with an altered topology of the outer membrane, particularly in regards to its Omps, which also serves to increase serum resistance (Caro-Hernández et al., 2007; Martín-Martín et al., 2011). The ability of rough *Brucella* spp. to exploit additional receptors, again, is unknown but is an intriguing possibility which might also explain their host specificity and organ tropism.

It seems clear that natural rough *Brucella* spp. have compensated for their lack of O-PS, as evidenced by their resistance to complement degradation, ability to replicate intracellularly, and lack of cytotoxicity and induction of proinflammatory cytokines. As with other Gram-negative bacteria, the rough phenotype might also serve to enhance the ability of rough *Brucella* spp. to avoid immune detection. Whether a rough LPS might also serve an advantage by increasing entry into macrophages or allowing the bacterium to use different receptors for cellular entry is not known but should be further explored, particularly in the context of the natural hosts.

## Smooth and Rough *Brucella*: Interaction With the Host Immune System

Although some differences do exist, interaction of natural rough *Brucella* spp. with host cells, including cellular entry, replication, and lack of proinflammatory cytokine production, closely resembles what occurs with smooth strains. These numerous similarities in host cell interactions suggest that components of the immune response engendered by both smooth and rough *Brucella* spp. may be similar, a concept which has significant implications for vaccine development. Work with *B. canis* and *B. ovis* in cells derived from the natural host and in the mouse model, described in the following section, may help to address this question.

### MHC-II Modulation

One mechanism in which smooth *Brucella* spp. interact with host cells to subvert the adaptive immune response is by inhibition of antigen presentation via Major Histocompatibility Complex (MHC)-II in macrophages. Macrophages utilize MHC-II to present antigen to CD4+ T lymphocytes, stimulating the adaptive immune response (Cresswell, 1994). The ability of *Brucella* spp. to interfere with this process is crucial to its long-term survival within the host, as IFN-γ-producing CD4+ T lymphocytes are a well-recognized requirement to controlling *Brucella* infection (Vitry et al., 2012). Interestingly, *Brucella* O-PS has also been found to interfere with this crucial branch between innate and adaptive immunity. One mechanism by which this occurs is disrupting the ability of MHC-II to present antigen following processing of LPS by macrophages. In this scenario, smooth LPS shed within the BCV is degraded by the macrophage followed by the formation of dense complexes, or macrodomains, which interfere with the interaction of MHC-II with CD4+ T lymphocytes through steric hindrance (Forestier et al., 1999, 2000). However, it appears that an additional mechanism for MHC modulation exists that is utilized by the natural rough strains as well. In a study using THP-1 cells, *B. abortus*, *B. ovis*, and heat-killed *B. abortus* inhibited IFN-γ-induced expression of MHC-II, suggesting the importance of some conserved element (Barrionuevo et al., 2008). Rather than LPS, Omp19 was the molecule responsible for this inhibition and this occurred in a TLR2-dependent manner (Barrionuevo et al., 2008). Thus, natural rough *Brucella* are capable of decreasing antigen presentation via MHC-II during the development of an adaptive immune response, independent of the structure of their LPS. Like the smooth strains, natural rough *Brucella* spp. can use MHC-II modulation to avoid detection by CD4+ T lymphocytes, promoting the chronic infections observed in their natural hosts.

### Interaction With Natural Host Cells

As professional antigen-presenting cells, dendritic cells play a key role in linking innate and adaptive immunity, and their role in the immune response to *Brucella* has been extensively evaluated. In terms of smooth *Brucella*, there remains some controversy as to whether infection of dendritic cells stimulates or inhibits their activation (Avila-Calderón et al., 2020). Overall, the majority of studies have shown that smooth *Brucella* spp. infection of dendritic cells results in downregulation of costimulatory markers (i.e., CD80 and CD86) and decreased production of proinflammatory cytokines while infection with rough mutants, such as RB51, results in the opposite effect (Zwerdling et al., 2008; Surendran et al., 2012; Avila-Calderón et al., 2020). The core oligosaccharide side branch, mentioned before for its importance in defense against complement and hampering of TLR4 recognition, appears to be important here as well. The *wadC* mutants of smooth strains, lacking this side branch, induce significant dendritic cell activation through recognition by TLR4 while their smooth parent strains do not (Conde-Álvarez et al., 2012). Comparatively little work on interaction with dendritic cells has been done with natural rough strains, but two studies by Pujol et al. (2017) have produced interesting findings concerning *B. canis*. These authors demonstrated that *B. canis* induces the expression of costimulatory molecules on both canine and human dendritic cells, but while infected human dendritic cells demonstrate TH1-skewed proinflammatory cytokine production, the canine cells show a mixed TH1-TH17 response (Pujol et al., 2017). This effect was also observed in CD4+ T lymphocytes stimulated by dendritic cells previously infected with *B. canis* (Pujol et al., 2019). These findings demonstrate that host specificity can play an important role in the immune response to *Brucella* spp.

A strong TH1 response, required for effective control of *Brucella* infection, by human dendritic cells could at least partially account for the lower susceptibility of humans to *B. canis* infection compared to the natural canine host. TH17 cells, on the other hand, have been shown to be associated with development of osteoarticular lesions in mice infected with *Brucella* spp., with IL-17 driving osteoclastogenesis (Giambartolomei et al., 2012). TH17 lymphocytes are also associated with autoimmune disorders and auto-antibody formation (Cornelius and Lamarca, 2014). The TH17 component of the response in canine dendritic cells, as suggested by Pujol et al. (2017), may reflect an increased susceptibility in this host and/or help to explain the incidence of osteoarticular lesions, particularly diskospondylitis, as well as antibody-mediated destruction of sperm in infected dogs (George and Carmichael, 1984; Pujol et al., 2019). Antibody-mediated attack on sperm is also a prominent feature of *B. ovis* infection in rams and the possible role of IL-17 remains to be explored in this species (Paolicchi et al., 2000). The association with IL-17 and disease in dogs infected with *B. canis* also warrants investigation. In addition to potential detrimental effects of IL-17 during *Brucella* infection, this cytokine appears to play some role in protective immunity early-on in the lungs following mucosal exposure (Mambres et al., 2016). The effect of IL-17 on defense against *Brucella* spp., smooth and rough, therefore appears to differ depending on the site of infection and deserves further study.

An additional host cell type that has been explored with *B. canis* infection is canine trophoblasts. Fernández et al. (2017) found that *B. canis* can infect and replicate within these cells or canine placental explants, causing no cytotoxicity but resulting in the secretion of IL-8 and RANTES (CCL5). Similar stimulation of IL-8 production has been seen in bovine placental explants, human trophoblasts, and human endometrial cell lines infected with *B. abortus* (Carvalho Neta et al., 2008; Fernández et al., 2016; Zavattieri et al., 2020). When the canine or human trophoblasts were exposed to culture supernatant from phagocytes infected with *B. canis* or *B. abortus*, the same stimulation of IL-8 was observed, suspected to be due to production of TNF-α by the phagocytes as has been shown to be the case in humans (Shimoya et al., 1999; Fernández et al., 2016, 2017). Increased IL-8 is expected to be responsible for drawing in neutrophils seen in the necrotizing placentitis characteristic in cases of infection with both *B. canis* in dogs or smooth *Brucella* spp. in their natural hosts (Fernández et al., 2017). Thus, it appears that both rough *B. canis* and the virulent smooth strains exhibit a similar pathogenesis in the female reproductive system in a still incompletely defined mechanism that is independent of O-PS. It remains to be seen how this cytokine induction compares with *B. ovis*, which is predominantly a pathogen of the male reproductive system with abortion being less common.

### Infection in the Mouse Model

Unlike many rough mutants, *B. ovis* and *B. canis* are able to establish and maintain a chronic, systemic infection in mice with organ distribution typical of smooth strains (Jiménez de Bagüés et al., 1993; Silva et al., 2011; Sá et al., 2012; Chacón-Díaz et al., 2015; Stranahan et al., 2019). However, the dose required to do so appears to be higher, at least for *B. canis*. For infection of mice with smooth *Brucella* spp., a dose of 10^4^–10^5^ CFU is typically used, resulting in early colonization of the spleen and liver with persistence in the spleen typically lasting longer than 36 weeks (High et al., 2007; Grilló et al., 2012). However, infection with *B. canis* at this dose in a previous study resulted in sporadic levels of colonization in the spleen with clearance achieved by 9 weeks (Stranahan et al., 2019). Vaccine studies in mice have noted levels of colonization in the spleen similar to or higher than the inoculation dose of 5 × 10^4^–5 × 10^5^, although these studies only examined a single time point post-infection and the full picture of the course of infection is difficult to ascertain in these cases (Edmonds et al., 2002; Clausse et al., 2014; Qian et al., 2017). Regardless, what is clear is that both *B. canis* and *B. ovis* appear to be less proinflammatory in mice than their smooth counterparts although it must be noted that in their natural hosts, both natural rough *Brucella* spp. can induce significant inflammation in target organs, including the epididymis (Carmichael and Kenney, 1968; Ficapal et al., 1998).

Splenomegaly is a classic feature of smooth *Brucella* spp. infection in mice (Enright et al., 1990; Grilló et al., 2012). In contrast, *B. canis* at doses up to 10^7^ CFU does not result in significant splenomegaly (Chacón-Díaz et al., 2015; Stranahan et al., 2019). This gross lesion may be induced at a high dose of 10^9^ CFU, but even then, the effect is transient (Stranahan et al., 2019). Interestingly, *B. ovis* does consistently appear to result in splenomegaly (Silva et al., 2011). *B. canis* also produces less significant histologic lesions in target organs, including fewer microgranulomas in the liver and histiocytic infiltrates in the spleen, than smooth strains despite identical inoculation doses and levels of colonization (Chacón-Díaz et al., 2015; Stranahan et al., 2019). Unexpectedly, *B. ovis* is able to induce numerous microgranulomas in the liver at 10^6^ CFU while such lesions are scarce in mice infected with the same dose of *B. canis*, emphasizing that while both rough, *B. ovis* and *B. canis* are by no means identical (Silva et al., 2011; Sá et al., 2012; Chacón-Díaz et al., 2015; Stranahan et al., 2019). In terms of cytokine induction *in vivo*, smooth strains are known to not cause significant induction of proinflammatory cytokines in mice (Barquero-Calvo et al., 2007). Nevertheless, both *B. canis* and *B. ovis* produce even lower amounts of such cytokines, including IFN-γ and IL-6, than smooth strains at the same dose (Sá et al., 2012; Chacón-Díaz et al., 2015). This is in contrast to the high levels of proinflammatory cytokines induced *in vitro* by rough mutants and it has been suggested, as mentioned above, that the lower levels induced by *B. canis* and *B. ovis* indicate that these strains exhibit an even stealthier intracellular life style (Rittig et al., 2003; Sá et al., 2012; Chacón-Díaz et al., 2015).

The distribution of *B. ovis* and *B. canis* to the spleen, liver, and lymph nodes in mice, as in their natural hosts, indicates that this laboratory animal can serve as a model for vaccine efficacy studies as it does for smooth strains (Carmichael and Kenney, 1968; Silva A. P. et al., 2015). Nevertheless, there are some limitations. For *B. ovis*, a significant cause of epididymitis in rams, one such deficiency is the lack of genital tropism or significant histologic lesions induced in the male reproductive system of mice (Silva et al., 2011). While *B. canis* is able to colonize the non-pregnant uterus of mice, it remains to be seen for both *B. ovis* and *B. canis* whether these natural rough strains can induce placentitis and fetal resorption noted with smooth strain infection in mice (Grilló et al., 2012).

The mouse model has been frequently employed to test vaccine candidates for *B. ovis* and less commonly for *B. canis*. For smooth strains, mice have been heavily utilized to investigate correlates of immune protection against infection, particularly in the context of vaccination. Such studies have established that control of smooth *Brucella* spp. infection is reliant on a strong TH1 immune response with IFN-γ representing the critical cytokine involved (Murphy et al., 2001; Baldwin and Goenka, 2006; Skendros and Boura, 2013). CD4+ and CD8+ T lymphocytes are the primary producers of IFN-γ in brucellosis and both appear to play key roles in the protective immune response, although the relative importance of each subset compared to the other remains controversial and appears to be influenced by route of infection and strain administered (Mambres et al., 2016; Pascual et al., 2019; Wang et al., 2020). For example, both CD4+ T lymphocytes and B lymphocytes are required to control infection with *B. melitensis* in mice following i.p. inoculation while only α/β T lymphocytes (either CD4+ or CD8+) are required for control following intranasal infection (Mambres et al., 2016; Demars et al., 2019). Comparatively little has been done to investigate the components of a protective immune response against the natural rough strains, particularly for *B. canis*. Nevertheless, protection against colonization induced by vaccination against both *B. canis* and *B. ovis* has been correlated with increased levels of IFN-γ production in mice (Cassataro et al., 2007b; Clausse et al., 2014; Eckstein et al., 2020; Moran et al., 2021). Additionally, as has been shown with *B. melitensis* and *B. abortus*, antibodies appear to play an important role in protection against *B. ovis* in mice (Jiménez de Bagüés et al., 1994; Bowden et al., 1995). Further investigation is required with the natural rough *Brucella* spp. to determine additional correlates of immune protection.

## Vaccine Development for Rough *Brucella* spp.

While the vaccines *B. abortus* S19, *B. abortus* RB51, and *B. melitensis* Rev.1 are commercially available to protect against infection by smooth *Brucella* spp. in livestock, no such vaccines exist for use in dogs. In addition, the Rev.1 vaccine, although protective against *B. ovis* in sheep, is not approved for use in areas free of *B. melitensis* (Blasco and Díaz, 1993; Ridler and West, 2011). Three questions may be asked in terms of the much-needed vaccine development for rough *B. ovis* and *B. canis*: (1) Is a live attenuated or killed/subunit vaccine superior? (2) Is the immune response required to protect against natural roughs by vaccination the same as that required against smooth strains? (3) Does it matter if a live attenuated vaccine (LAV) to protect against the natural roughs is smooth or rough and will this impact cross-protection against smooth strains of *Brucella* in the vaccinated animal? The first question is still debated even for the smooth *Brucella* spp., although a preponderance of evidence from the natural host indicates that LAVs provide the greatest levels of protection against brucellosis due to their ability to generate more persistent memory responses (Ficht et al., 2009; Perkins et al., 2010). When comparing the protection indices of studies investigating vaccine candidates against *B. ovis* and *B. canis*, as measured by differences in log_10_ colonization of the spleen between vaccinated and unvaccinated animals, the picture is less clear (Tables 2, 3). Higher protection indices are typically noted for LAVs than for subunit vaccines, although there is frequent overlap and many instances in which protection afforded by subunit vaccines approaches or equals that offered by LAVs. Work with *B. canis* has highlighted that the choice of adjuvant can significantly impact the efficacy of a subunit vaccine (Clausse et al., 2014) and studies involving both natural rough strains have demonstrated improved protection of certain LAVs when given in an encapsulated form, as has been shown for smooth *Brucella* spp. (Ficht et al., 2009; Silva A. P. et al., 2015; Eckstein et al., 2020).

**TABLE 2:** Vaccine candidates against *B. ovis* investigated in the mouse model in select studies.

Candidate vaccine	Route/dose	Mouse strain	Challenge strain, dose/route	# vaccinations	Protection index	References
*B. ovis*Δ*abcBA*	10^8^ CFU, s.q., alginate-encapsulated	BALB/c	*B. ovis* ATCC 25840, 10^6^ CFU, i.p.	1	0.54	Silva T. M. et al., 2015
		C57BL/6			1.01	
*B. ovis*Δ*omp25d*	10^7^ CFU, i.p.	BALB/c	*B. ovis* PA, 10^5^ CFU, i.p.	1	∼3.6	Sancho et al., 2014
*B. ovis*Δ*omp22*					∼3	
Rev.1	10^5^ CFU, i.p.				∼3.4	
*B. ovis*Δ*wadB*	10^8^ CFU, i.p.	BALB/c	*B. ovis* PA, 5 × 10^5^ CFU, i.p.	1	5.16	Soler-Lloréns et al., 2014
*B. ovis*Δ*wadC*					4.06	
Rev.1	10^5^ CFU, s.q.				3.49	
*B. melitensis* BM 115	10^8^ CFU, i.p.	BALB/c	*B. ovis* 63/290, 2 × 10^8^ CFU, i.p.	1	3	Adone et al., 2008
Rev.1	10^8^ CFU, i.p.				3.6	
Rev.1 Δ*bp26*	5 × 10^4^ CFU, s.q.	BALB/c	*B. ovis* PA, 5 × 10^5^ CFU, i.p.	1	2.44	Cloeckaert et al., 2004
Rev.1 Δ*omp31*					3.49	
Rev.1 Δ*bp26*Δ*omp31*					2.56	
Rev.1					2.64	
*B. abortus*Δ*wbkA*	10^8^ CFU, i.p.	BALB/c	*B. ovis* PA, 8 × 10^4^ CFU, i.p.	1	4.6	Monreal et al., 2003
*B. abortus*Δ*per*					2.86	
*B. abortus*Δ*manB*					0	
B. abortus Δwa**					1.14	
RB51					3.11	
S19	10^5^ CFU, s.q.				3.12	
RB51	3 × 108 CFU, i.p.	BALB/c	*B. ovis* PA	1	2.3	Jiménez de Bagüés et al., 1994
BLSOmp31	30 μg, s.q., + CpG-ODN + Coa-ASC16	BALB/c	*B. ovis* PA, 2.9 × 10^5^ CFU, i.p.	2	3.61	Moran et al., 2021
HS extract (*B. ovis* REO198)	12 μg, conjunctival, mannosylated nanoparticles.	BALB/c	*B. ovis*, 510^4^ CFU, i.p.	1	2.93	Da Costa Martins et al., 2012
Rev.1	5 × 10^5^ CFU, s.q.				2.52	
pcDNABLS	100 μg, i.m.	BALB/c	*B. ovis*, 10^4^ CFU, i.v.	4	1.94	Cassataro et al., 2007a
pCIOmp31					2.24	
pcDNABLS + pCIOmp31					2.20	
pCIBLSOmp31					3.14	
Rev.1	2.2 × 10^4^ CFU, s.q.			1	2.42	
BLS	30 μg, s.q., + IFA	BALB/c	*B. ovis*, 10^4^ CFU, i.v.	2	1.29	Cassataro et al., 2007b
Omp31					1.98	
BLSOmp31					2.69	
Rev.1	2.2 × 10^4^ CFU, s.q.			1	2.41	
Omp31	20 μg, i.p.	BALB/c	*B. ovis* PA, 1.6 × 10^4^ CFU, i.p.	2	2.06	Estein et al., 2003
R-LPS					1.63	
Omp31 + R-LPS					2.26	
HS extract					2.57	

*White cells indicate studies involving live attenuated vaccine (LAV) candidates while gray cells indicate those associated with subunit/killed candidates.*

**TABLE 3:** Vaccine candidates against *B. canis* investigated in the mouse model in select studies.

Candidate vaccine	Route/dose	Mouse strain	Challenge strain, dose/route	# vaccinations	Protection index	References
*B. ovis*Δ*abcBA*	10^8^ CFU, s.q., alginate-encapsulated	BALB/c	*B. canis* ATCC 23365, 10^6^ CFU, i.p.	1	1.5	Eckstein et al., 2020
*B. canis ΔvjbR*	3 × 10^7^ CFU, i.p.	BALB/c	*B. canis* RM6/66, 10^7^ CFU, i.p.	1	2.98	Liu et al., 2020
A19	10^5^ CFU, i.p.			1	3.43	
*B. canis*Δ*vjbR*	10^9^ CFU, s.q.	C57BL/6J	*B. canis* RM6/66, 10^7^ CFU, i.p.	1	4.14	Stranahan et al., 2020
RB51	3.9–4.8 × 10^8^ CFU, i.p.	BALB/c	*B. canis* S26, 2.8 × 10^5^ CFU, i.p.	1	1.56	Truong et al., 2015
RB51Δ*cydC*				2	2.03	
RB51Δ*cydD*				2	2.11	
RB51Δ*purD*				2	2.20	
*B. canis*Δ*virB10*	1.4 × 10^8^ CFU, i.p.	BALB/c	*B. canis*, 5 × 10^4^ CFU, i.p.	1	1.91	Palomares-Resendiz et al., 2012
*B. canis*Δ*virB11*				1	1.96	
*B. ovis*Δ*omp25*	5 × 10^4^ CFU i.v.	BALB/c	*B. canis* RM6/66, 5 × 10^4^ CFU, i.v.	1	1.15	Edmonds et al., 2002
*B. melitensis*Δ*omp25*	5 × 10^4^ CFU i.v.			1	1.62	
Rev.1	5 × 10^4^ CFU i.v.			1	1.95	
RB51	3 × 10^8^ CFU, i.p.			1	0.46	
*B. canis* ghost	10^8^ CFU, i.p.	BALB/c	*B. canis* RM6/66, 5x10^5^ CFU, i.p.	1	2.37	Qian et al., 2017
rOmp31	30 μg, s.q., + montanide	BALB/c	*B. canis* RM6/66, 5.5 × 10^5^ CFU, i.p.	2	1.42	Clausse et al., 2014
	30 μg, s.q., + Quil-A				1.86	
	30 μg, s.q., + IFA				1.66	
	30 μg, s.q., + AH				1.65	
Heat-killed *B. canis*	10^9^ CFU, s.q. + IFA				3.48	
pCIBLSOmp31 + rOmp31	100 μg, 30 μg, i.m.	BALB/c	*B. canis* RM6/66, 5.5 × 10^5^ CFU, i.p.	3,1	2.29	Clausse et al., 2013
BLSOmp31	30 μg, s.q. + AH			2	1.57	
	30 μg, s.q. + IFA				4.02	
	30 μg, s.q. + montanide				0.36	
	30 μg, s.q. + Quil-A				1.54	
*B. canis* bacterin	6.3 × 10^8^ CFU, s.q. + IFA				4.38	
*B. ovis*	10^9^ CFU, i.p.			1	4.12	

*White cells indicate studies involving live attenuated vaccine (LAV) candidates while gray cells indicate those associated with subunit/killed candidates.*

As for the components of a protective immune response against rough *Brucella* spp., what is so far known from research in mice, as indicated in the preceding section, appears to reflect what is observed for smooth strains. In the few vaccine studies which have examined it, IFN-γ is higher in stimulated splenocytes in groups of mice that exhibit greater levels of protection against both *B. ovis* and *B. canis* (Cassataro et al., 2005b, 2007b; Truong et al., 2015; Eckstein et al., 2020; Moran et al., 2021). There is also limited evidence that antibodies play a role in protection against *B. ovis* in vaccinated mice (Jiménez de Bagüés et al., 1994). For the natural dog and ovine hosts, a TH1 response also appears critical for protection. In dogs, the current understanding is that animals which achieve self-elimination of *B. canis*, at least to below the level of detection by culture, are immune to subsequent challenge secondary to a strong cell-mediated immune response (Carmichael and Greene, 2006; Cosford, 2018). In contrast, persistently infected dogs exhibit a stronger humoral immune response and these dogs that undergo treatment with antibiotics are fully susceptible to secondary challenge (Carmichael and Greene, 2006). For sheep, the relative importance of humoral immunity is less certain but TH1/cell-mediated responses with high levels of IFN-γ are associated with higher levels of protection against *B. ovis* in Rev.1- or subunit-vaccinated animals (Estein et al., 2009; Galindo et al., 2009).

While CD4+ T lymphocytes were long considered the most important source of IFN-γ in the response against brucellosis, several recent studies have indicated that CD8+ T lymphocytes may be equally or sometimes more important in mediating protection depending on the vaccine administered. For example, one study found that CD4+ T lymphocytes were required for protection against *B. melitensis* in mice following vaccination with RB51 while CD8+ T lymphocytes were required following vaccination with the candidate *B. abortus znBAZ* (Wang et al., 2020). Whether this is related to the rough phenotype of RB51 or separate properties of this LAV is uncertain. For the natural rough *Brucella* spp., such aspects of the immune response during vaccination have been little explored but also appear to be influenced by the type of vaccine. In work investigating the protective efficacy of Omp31 as a vaccine against *B. ovis* in mice, protection afforded by administration of the recombinant protein was mediated by CD4+ T lymphocytes while protection acquired through DNA coding for Omp31 was mediated mainly by CD8+ T lymphocytes (Cassataro et al., 2005a, b). It is clear that further work needs to be done to investigate components of a protective immune response against natural rough strains induced by vaccination, particularly for *B. canis*. An additional need is to perform these investigations using a natural, mucosal route of infection rather than the convenient but artificial intraperitoneal (i.p.) route. This concern has become apparent recently as studies with smooth *Brucella* spp. indicate that correlates of immune protection differ depending on route of infection, with B cells, for instance, being required for protection in mice following i.p. inoculation but being dispensable following intranasal infection (Mambres et al., 2016; Demars et al., 2019).

Finally, should a vaccine against *B. ovis* or *B. canis* be produced in a smooth or rough background and will this impact cross-protection against smooth strains? This question is critical as both sheep and dogs are susceptible to infection by smooth *Brucella* spp., including *B. melitensis* for sheep and *B. suis* for dogs (Pappas et al., 2006; Ramamoorthy et al., 2011). Unlike the vaccines S19 and Rev.1, RB51 is a stable rough vaccine. While its LPS phenotype putatively results in less interference with serologic assays, RB51 possesses the disadvantages of antibiotic resistance, virulence for humans, and decreased efficacy compared to S19 (Winter et al., 1996; Ficht et al., 2009). Interestingly, while RB51 provides protection against *B. ovis* in mice, it is not likewise effective in sheep (Jiménez de Bagüés et al., 1994, 1995). Of note, this study suggested through passive transfer that the protection afforded against *B. ovis* in mice was predominantly mediated by the humoral immune response while protection against *B. abortus* was mainly cell-mediated, although this protection was much lower than that afforded by the vaccine (Jiménez de Bagüés et al., 1994). RB51 is also able to provide similar levels of protection against *B. canis* and *B. abortus* in mice (Truong et al., 2015). Efficacy of RB51 in dogs is uncertain and although it is not shed in the urine or feces of vaccinated dogs, it can colonize various organs, including the placenta, which could serve as a source of infection to humans or other animals and therefore precludes its use in dogs (Palmer and Cheville, 1997).

The deficiencies of RB51 do not mean that rough vaccines cannot be protective against infection by *Brucella* spp., rough or smooth. RB51’s reduced efficacy against *B. ovis* and its smooth counterparts in the natural host may be associated with its attenuation and/or alterations to its outer membrane or LPS outside of its lack of O-PS. Several studies have demonstrated that targeted mutagenesis can produce rough vaccines that are superior in protection to RB51 and comparable to protection by S19 or Rev.1 in mice ([Bibr B114][Bibr B90]; Aragón-Aranda et al., 2020). One prominent finding is that for a rough vaccine to be effective, the core must be intact although lack of the aforementioned lateral side branch may actually enhance vaccine efficacy, presumably due to enhanced recognition of surface molecules in the absence of this “shield” (Monreal et al., 2003; Soler-Lloréns et al., 2014). Additionally, while some rough vaccine candidates are not as protective against smooth *Brucella* spp. as is S19 in mice, the difference may largely be accounted for by the activity of anti-O-PS antibodies, a component of the immune response that appears important in mice (depending on route of infection) but is of controversial significance in the natural hosts (Monreal et al., 2003). In *B. ovis* vaccine studies, the rough vaccine *B. melitensis* 115 (*wzm* mutation) provides similar levels of protection against both *B. melitensis* and *B. ovis* while *B. ovis ΔabcBA* affords comparable protection against *B. melitensis, B. ovis*, and *B. canis* (Adone et al., 2008; Costa et al., 2020; Eckstein et al., 2020). Nevertheless, some caveats must be pointed out. First, certain rough LAV candidates such as *B. melitensis* B115 still produce partial or complete O-PS that remains within the cytoplasm and the possibility that this internal O-PS assists in mediating protection cannot be ruled out. Second, some studies administered rough vaccine candidates at a higher dose than smooth candidates, as is the case with the recommended doses of RB51 vs. S19 in mice (10^8^ CFU vs. 10^5^ CFU) (Adone et al., 2008, 2011; Aragón-Aranda et al., 2020). Despite this, these studies, although still few in number, indicate that a rough vaccine has the potential to protect against both smooth and rough *Brucella* spp. and deserve further investigation. Advantages would include the oft-cited lack of interference with current serologic assays detecting anti-O-PS antibodies in addition to the possibility of producing an LAV on a background strain that is either non-zoonotic (*B. ovis*) or exhibits reduced virulence for humans (*B. canis*).

As is known for development of vaccines against smooth *Brucella* spp., there are limitations to the mouse model for development of *B. ovis* and *B. canis* vaccines and there is a significant need for more work in the natural hosts for these rough organisms, particularly *B. canis*. As mentioned above, RB51 is moderately protective against *B. ovis* in mice but has no efficacy in sheep likely due to as yet incompletely understood differences in the immune response and/or differences in challenge route utilized between these two hosts (Jiménez de Bagüés et al., 1994, 1995). On the other hand, certain vaccines such as *B. ovis*Δ*abcBA* provide minimal protection in the mouse but are highly effective against experimental *B. ovis* infection in rams (Silva A. P. et al., 2015; Silva T. M. et al., 2015). Of additional concern, while Rev.1 can serve as a reference vaccine for comparison purposes in *B. ovis* vaccine studies, no such reference is available for *B. canis*.

## Concluding Remarks

Conspicuously lacking in O-PS, *B. canis* and *B. ovis* are virulent pathogens in their natural hosts that are able to recapitulate many of the properties and cellular interactions observed by their more famous smooth cousins. The lack of a vaccine for dogs and the recent rise in detection of *B. canis* infection along with the negative economic impact of *B. ovis* on sheep production necessitates a more thorough understanding of these rough organisms and their interactions with the host. Oftentimes in complete contrast to what occurs with rough mutants of smooth *Brucella* spp., these natural rough strains are able to evade and manipulate the host immune system by exhibiting low endotoxic activity, resisting destruction by complement and antimicrobial peptides, entering and trafficking within host cells along a similar pathway, and interfering with MHC-II antigen presentation. *B. canis* and *B. ovis* appear to have compensated for the loss of O-PS by alterations to their outer membrane, particularly in regards to Omps. Whether this loss also serves an evolutionary advantage, as it does for other rough Gram-negative bacteria such as *Y. pestis*, is uncertain although the lower proinflammatory profile induced *in vitro* and in mice is suggestive of an enhanced level of stealth that could allow these pathogens to persist for long periods of time undetected. Given the commonalities in behavior between natural rough and smooth *Brucella* spp., it is not surprising that the immune response required to achieve protection against either phenotype appears similar. Nevertheless, much additional work is required to understand the correlates of immune protection against the natural rough *Brucella* spp., both in the natural host and involving mucosal routes of infection. Finally, the mouse model is useful in supporting the continued search for a vaccine against *B. canis* and evidence so far points to the superior protection of LAVs. Despite RB51’s reduced efficacy compared to S19 and Rev.1, there is evidence that a rough vaccine could serve to protect sheep and dogs against not only *B. ovis* and *B. canis*, but against smooth strains as well.

## Author Contributions

LS and AA-G contributed to the conception of the review. LS drafted the manuscript. Both authors reviewed and approved the manuscript.

## Conflict of Interest

The authors declare that the research was conducted in the absence of any commercial or financial relationships that could be construed as a potential conflict of interest.

## References

[B1] Adone R. Francia M. Ciuchini F. (2008). Evaluation of *Brucella melitensis* B115 as rough-phenotype vaccine against *B. melitensis* and *B. ovis* infections. *Vaccine* 26 4913–4917. 10.1016/j.vaccine.2008.07.030 18675869

[B2] Adone R. Francia M. Pistoia C. Pesciaroli M. Pasquali P. (2011). *B. melitensis* rough strain B115 is protective against heterologous *Brucella* spp. infections. *Vaccine* 29 2523–2529. 10.1016/j.vaccine.2011.01.072 21300102

[B3] Allen C. A. Adams L. G. Ficht T. A. (1998). Transposon-derived *Brucella abortus* rough mutants are attenuated and exhibit reduced intracellular survival. *Infect. Immun.* 66 1008–1016. 10.1128/IAI.66.3.1008-1016.1998 9488389 PMC108009

[B4] Alton G. G. Jones L. M. Angus R. D. Verger J. M. (1988). Techniques for the brucellosis laboratory. *Br. Vet. J.* 146:188. 10.1016/0007-1935(90)90017-W

[B5] Aragón-Aranda B. de Miguel M. J. Lázaro-Antón L. Salvador-Bescós M. Zúñiga-Ripa A. Moriyón I. (2020). Development of attenuated live vaccine candidates against swine brucellosis in a non-zoonotic *B. suis* biovar 2 background. *Vet. Res.* 51:92. 10.1186/s13567-020-00815-8 32703299 PMC7376850

[B6] Avila-Calderón E. Flores-Romo L. Sharon W. Donis-Maturano L. Becerril-García M. (2020). Dendritic cells and *Brucella* spp. interaction: the sentinel host and the stealthy pathogen. *Folia Microbiol.* 65 1–16. 10.1007/s12223-019-00691-6 30783994 PMC7224029

[B7] Baldi P. C. Giambartolomei G. H. (2013). Pathogenesis and pathobiology of zoonotic brucellosis in humans. *Rev. Sci. Tech.* 32 117–125. 10.20506/rst.32.1.2192 23837370

[B8] Baldwin C. L. Goenka R. (2006). Host immune responses to the intracellular bacteria *Brucella*: Does the bacteria instruct the host to facilitate chronic infection? *Crit. Rev. Immunol.* 26 407–442. 10.1615/critrevimmunol.v26.i5.30 17341186

[B9] Barquero-Calvo E. Chaves-Olarte E. Weiss D. S. Guzmán-Verri C. Chacón-Díaz C. Rucavado A. (2007). *Brucella abortus* uses a stealthy strategy to avoid activation of the innate immune system during the onset of infection. *PLoS One* 2:e631. 10.1371/journal.pone.0000631 17637846 PMC1910614

[B10] Barrionuevo P. Cassataro J. Delpino M. V. Zwerdling A. Pasquevich K. A. Samartino C. G. (2008). *Brucella abortus* inhibits major histocompatibility complex class II expression and antigen processing through interleukin-6 secretion via Toll-like receptor 2. *Infect. Immun.* 76 250–262. 10.1128/IAI.00949-07 17984211 PMC2223644

[B11] Baucheron S. Grayon M. Zymgunt M. S. Cloeckaert A. (2002). Lipopolysaccharide heterogeneity in *Brucella* spp. isolated from marine mammals. *Res. Microbiol.* 153 277–280. 10.1016/s0923-2508(02)01317-7 12160318

[B12] Billard E. Dornand J. Gross A. (2007). Interaction of *Brucella suis* and *Brucella abortus* rough strains with human dendritic cells. *Infect. Immun.* 75 5916–5923. 10.1128/IAI.00931-07 17938225 PMC2168365

[B13] Blasco J. M. (1990). “Brucella ovis,” in *Animal Brucellosis*, eds Nielsen K. Duncan J. R. (Boca Raton, FL: CRC Press, Inc), 352–378.

[B14] Blasco J. M. Díaz R. (1993). *Brucella melitensis* Rev-1 vaccine as a cause of human brucellosis. *Lancet* 342:805. 10.1016/0140-6736(93)91571-3 8103891

[B15] Bowden R. A. Cloeckaert A. Zygmunt M. S. Dubray G. (1995). Outer-membrane protein- and rough lipopolysaccharide-specific monoclonal antibodies protect mice against *Brucella ovis*. *J. Med. Microbiol.* 43 344–347. 10.1099/00222615-43-5-344 7562998

[B16] Bricker B. Goonesekere N. Bayles D. Alt D. Olsen S. Vrentas C. (2020). Genome report- a genome sequence analysis of the RB51 strain of *Brucella abortus* in the context of its vaccine properties. *G3* 10 1175–1181. 10.1534/g3.119.400964 32111651 PMC7144086

[B17] Buddle M. B. (1956). Studies on *Brucella ovis* (n.sp.), a cause of genital disease of sheep in New Zealand and Australia. *J. Hyg.* 54 351–364. 10.1017/s0022172400044612 13367402 PMC2217862

[B18] Cardoso P. G. Macedo G. C. Azevedo V. Oliveira S. C. (2006). *Brucella* spp noncanonical LPS: structure, biosynthesis, and interaction with host immune system. *Microb. Cell Fact.* 5:13. 10.1186/1475-2859-5-13 16556309 PMC1435926

[B19] Carmichael L. E. Greene C. E. (2006). “Canine brucellosis,” in *Infectious Diseases of the Dog and Cat*, ed. Greene C. E. (Philadelphia, PA: Elsevier), 369–381.

[B20] Carmichael L. E. Kenney R. M. (1968). Canine abortion caused by *Brucella canis*. *J. Am. Vet. Med. Assoc.* 152 605–616. 5688953

[B21] Caro-Hernández P. Fernández-Lago L. de Miguel M. J. Martín-Martín A. I. Cloeckaert A. Grilló M. J. (2007). Role of the Omp25/Omp31 family in outer membrane properties and virulence of *Brucella ovis*. *Infect. Immun.* 75 4050–4061. 10.1128/IAI.00486-07 17562767 PMC1952020

[B22] Carvalho Neta A. V. Stynen A. P. Paixão T. A. Miranda K. L. Silva F. L. Ruox C. M. (2008). Modulation of the bovine trophoblastic innate immune response by *Brucella abortus*. *Infect. Immun.* 76 1897–1907. 10.1128/IAI.01554-07 18316388 PMC2346690

[B23] Cassataro J. Estein S. M. Pasquevich K. A. Velikovsky C. A. de la Barrera S. Bowden R. (2005a). Vaccination with the recombinant *Brucella* outer membrane protein 31 or a derived 27-amino-acid synthetic peptide elicits a CD4+ T helper 1 response that protects against *Brucella melitensis* infection. *Infect. Immun.* 73 8079–8088. 10.1128/IAI.73.12.8079-8088.2005 16299302 PMC1307072

[B24] Cassataro J. Pasquevich K. A. Estein S. M. Laplagne D. A. Velikovsky C. A. de la Barrera S. (2007a). A recombinant subunit vaccine based on the insertion of 27 amino acids from Omp31 to the N-terminus of BLS induced as similar degree of protection against *B. ovis* than Rev.1 vaccination. *Vaccine* 25 4437–4446. 10.1016/j.vaccine.2007.03.028 17442465

[B25] Cassataro J. Pasquevich K. A. Estein S. M. Laplagne D. A. Zwerdling A. de la Barrera S. (2007b). A DNA vaccine coding for the chimera BLSOmp31 induced a better degree of protection against *B. ovis* and a similar degree of protection against *B. melitensis* than Rev.1 vaccination. *Vaccine* 25 5958–5967. 10.1016/j.vaccine.2007.05.049 17600596

[B26] Cassataro J. Velikovsky C. A. de la Barrera S. Estein S. M. Bruno L. Bowden R. (2005b). A DNA vaccine coding for the *Brucella* outer membrane protein 31 confers protection against *B. melitensis* and *B. ovis* infection by eliciting a specific cytotoxic response. *Infect. Immun.* 73 6537–6546. 10.1128/iAI/73.10.6537-6546.2005 16177328 PMC1230944

[B27] Celli J. (2015). The changing nature of the *Brucella*-containing vacuole. *Cell. Microbiol.* 17 951–958. 10.1111/cmi.12452 25916795 PMC4478208

[B28] Chacón-Díaz C. Altamirano-Silva P. González-Espinoza G. Medina M. C. Alfaro-Alarcón A. Bouza-Mora L. (2015). *Brucella canis* is an intracellular pathogen that induces a lower proinflammatory response than smooth zoonotic counterparts. *Infect. Immun.* 83 4861–4870. 10.1128/IAI.00995-15 26438796 PMC4645416

[B29] Chen F. He Y. (2009). Caspase-2-mediated apoptotic and necrotic murine macrophage cell death induced by rough *Brucella abortus*. *PLoS One* 4:e6830. 10.1371/journal.pone.0006830 19714247 PMC2729395

[B30] Clausse M. Díaz A. G. Ghersi G. Zylberman V. Cassataro J. Giambartolomei G. H. (2013). The vaccine candidate BLSOmp31 protects mice against *Brucella canis* infection. *Vaccine* 31 6129–6135. 10.1016/j.vaccine.2013.07.041 23906889

[B31] Clausse M. Díaz A. G. Ibañez A. E. Cassataro J. Giambartolomei G. H. Estein S. M. (2014). Evaluation of the efficacy of outer membrane protein 31 vaccine formulations for protection against *Brucella canis* in BALB/c mice. *Clin. Vaccine Immunol.* 21 1689–1694. 10.1128/CVI.00527-14 25339409 PMC4248782

[B32] Cloeckaert A. Jacques I. Grilló M. J. Marín C. M. Grayon M. Blasco J. M. (2004). Development and evaluation as vaccines in mice of *Brucella melitensis* Rev.1 single and double deletion mutants of the *bp26* and *omp31* genes coding for antigens of diagnostic significance in ovine brucellosis. *Vaccine* 22 2827–2835. 10.1016/j.vaccine.2004.01.001 15246618

[B33] Cloeckaert A. Vizcaíno N. Paquet J. Bowden R. A. Elzer P. H. (2002). Major outer membrane proteins of *Brucella* spp.: past, present, and future. *Vet. Microbiol.* 90 229–247. 10.1016/s0378-1135(02)00211-0 12414146

[B34] Cloeckaert A. Weynants V. Godfroid J. Verger J. M. Grayon M. Zygmunt M. S. (1998). O-polysaccharide epitopic heterogeneity at the surface of *Brucella* spp. studied by enzyme-linked immunosorbent assay and flow cytometry. *Clin. Diagn. Lab. Immunol.* 5 862–870. 10.1128/CDLI.5.6.862-870.1998 9801349 PMC96216

[B35] Conde-Álvarez R. Arce-Gorvel V. Iriarte M. Mancek-Keber M. Barquero-Calvo R. Palacios-Chaves L. (2012). The lipopolysaccharide core of *Brucella abortus* acts as a shield against innate immunity recognition. *PLoS Pathog.* 8:e1002675. 10.1371/journal.ppat.1002675 22589715 PMC3349745

[B36] Connor M. G. Pulsifer A. R. Chung D. Rouchka E. C. Ceresa B. K. Lawrenz M. B. (2018). Yersinia pestis targets the host endosome recycling pathway during the biogenesis of the Yersinia-containing vacuole to avoid killing by macrophages. *mBio* 9:e01800-17. 10.1128/mBio.01800-17 29463656 PMC5821078

[B37] Corbeil L. B. Blau K. Inzana T. J. Nielsen K. H. Jacobson R. H. Corbeil R. R. (1988). Killing of *Brucella abortus* by bovine serum. *Infect. Immun.* 56 3251–3261. 10.1128/IAI.56.12.3251-3261.1988 3141287 PMC259732

[B38] Corbel M. J. (1997). Brucellosis: an overview. *Emerg. Infect. Dis.* 3 213–221. 10.3201/eid0302.970219 9204307 PMC2627605

[B39] Cornelius D. C. Lamarca B. (2014). TH17- and IL-17-mediated autoantibodies and placental oxidative stress play a role in the pathophysiology of pre-eclampsia. *Minerva Ginecol.* 66 243–249. 24971780 PMC5089699

[B40] Cosford K. L. (2018). *Brucella canis*: an update on research and clinical management. *Can. Vet. J.* 59 74–81. 29302106 PMC5731389

[B41] Costa L. C. Cabello A. L. Batista D. F. A. Chaki S. P. de Figeuiredo P. da Paixão T. A. (2020). The candidate vaccine strain *Brucella ovis* ΔabcBA is protective against *Brucella melitensis* infection in mice. *Microbiol. Immunol.* 64 730–736. 10.1111/1348-0421.12850 32965738

[B42] Cresswell P. (1994). Assembly, transport, and function of MHC class II molecules. *Annu. Rev. Immunol.* 12 259–293. 10.1146/annurev.iy.12.040194.001355 8011283

[B43] Da Costa Martins R. Gamazo C. Sánchez-Martín M. Barberán M. Peñuelas I. Irache J. M. (2012). Conjunctival vaccination against *Brucella ovis* in mice with mannosylated nanoparticles. *J. Control. Release* 162 553–560. 10.1016/j.jconrel.2012.07.030 22846987

[B44] Delpino M. V. Fossati C. A. Baldi P. C. (2009). Proinflammatory response of human osteoblastic cell lines and osteoblast-monocyte interaction upon infection with *Brucella* spp. *Infect. Immun.* 77 984–995. 10.1128/IAI.01259-08 19103778 PMC2643642

[B45] Demars A. Lison A. Machelart A. Van Vyve M. Potemberg G. Vanderwinden J. M. (2019). Route of infection strongly impacts the host-pathogen relationship. *Front. Immunol.* 10:1589. 10.3389/fimmu.2019.01589 31354728 PMC6637429

[B46] Detilleux P. G. Deyoe B. L. Cheville N. F. (1990). Entry and intracellular localization of *Brucella* spp. in Vero cells: fluorescence and electron microscopy. *Vet. Pathol.* 27 317–328. 10.1177/030098589002700503 2122572

[B47] Dolan B. Naughton J. Tegtmeyer N. May F. E. Clyne M. (2012). The interaction of *Helicobacter* pylori with the adherent mucus gel layer secreted by polarized HT29-MTX-E12 cells. *PLoS One* 7:e47300. 10.1371/journal.pone.0047300 23056622 PMC3466223

[B48] Dornand J. Lafont V. Oliaro J. Terraza A. Castañeda-Roldán E. Liautard J. P. (2004). Impairment of intramacrophagic *Brucella suis* multiplication by human natural killer cells through a contact-dependent mechanism. *Infect. Immun.* 72 2303–2311. 10.1128/iai.72.4.2303-2311.2004 15039355 PMC375199

[B49] Dueñas A. I. Orduña A. Crespo M. S. García-Rodríguez C. (2004). Interaction of endotoxins with Toll-like receptor 4 correlates with their endotoxic potential and may explain the proinflammatory effect of *Brucella* spp. LPS. *Int. Immunol.* 16 1467–1475. 10.1093/intimm/dxh148 15339879

[B50] Eckstein C. Mol J. P. Costa F. B. Nunes P. P. Lima P. A. Melo M. M. (2020). *Brucella ovis* mutant in ABC transporter protects against *Brucella canis* infection in mice and it is safe for dogs. *PLoS One* 15:e0231893. 10.1371/journal.pone.0231893 32298378 PMC7162469

[B51] Edmonds M. D. Cloeckaert A. Elzer P. H. (2002). *Brucella* species lacking the major outer membrane protein Omp25 are attenuated in mice and protect against *Brucella melitensis* and *Brucella ovis*. *Vet. Microbiol.* 88 205–221. 10.1016/s0378-1135(02)00110-4 12151196

[B52] Eisenschenk F. C. Houle J. J. Hoffman E. M. (1999). Mechanism of serum resistance among *Brucella abortus* isolates. *Vet. Microbiol.* 68 235–244. 10.1016/s0378-1135(99)00075-9 10510042

[B53] Enright F. M. Araya L. N. Elzer P. H. Rowe G. E. Winter A. J. (1990). Comparative histopathology in BALB/c mice infected with virulent and attenuated strains of *Brucella abortus*. *Vet. Immunol. Immunopathol.* 26 171–182. 10.1016/0165-2427(90)90065-z 2124401

[B54] Erridge C. Bennet-Guerrero E. Poxton I. R. (2002). Structure and function of lipopolysaccharides. *Microbes Infect.* 4 837–851. 10.1016/s1286-4579(02)01604-0 12270731

[B55] Eskra L. Covert J. Glasner J. Splitter G. (2012). Differential expression of iron acquisition genes by *Brucella melitensis* and *Brucella canis* during macrophage infection. *PLoS One* 7:e31747. 10.1371/journal.pone.0031747 22403618 PMC3293887

[B56] Estein S. M. Cassataro J. Vizcaíno N. Zygmunt M. Cloeckaert A. Bowden R. A. (2003). The recombinant Omp31 from *Brucella melitensis* alone or associated with rough lipopolysaccharide induces protection against *Brucella ovis* infection in BALB/c mice. *Microbes Infect.* 5 85–93. 10.1016/s1286-4579(02)00075-8 12650766

[B57] Estein S. M. Fiorentino M. A. Paolicchi F. A. Clausse M. Manazza J. Cassataro J. (2009). The polymeric antigen BLSOmp31 confers protection against *Brucella ovis* in rams. *Vaccine* 27 6704–6711. 10.1016/j.vaccine.2009.08.097 19748579

[B58] Evans E. Poxton I. R. Govan J. W. (1999). Lipopolysaccharide chemotypes in *Burkholderia cepacian*. *J. Med. Microbiol.* 48 825–832. 10.1099/00222615-48-9-825 10482293

[B59] Fernández A. G. Ferrero M. C. Hielpos M. S. Fossati C. A. Baldi P. C. (2016). Proinflammatory response of human trophoblastic cells to *Brucella abortus* infection and upon interactions with infected phagocytes. *Biol. Reprod.* 94:48. 10.1095/biolreprod.115.131706 26792938

[B60] Fernández A. G. Hielpos M. S. Ferrero M. C. Fossati C. A. Baldi P. C. (2017). Proinflammatory response of canine trophoblasts to *Brucella canis* infection. *PLoS One* 12:e0186561. 10.1371/journal.pone.0186561 29036184 PMC5643107

[B61] Fernandez-Prada C. M. Zelazowska E. B. Nikolich M. Hadfield T. L. Roop R. M. Robertson G. L. (2003). Interactions between *Brucella melitensis* and human phagocytes: bacterial surface O-polysaccharide inhibits phagocytosis, bacterial killing, and subsequent host cell apoptosis. *Infect. Immun.* 71 2110–2119. 10.1128/iai.71.4.2.2110-2119.2003 12654833 PMC152029

[B62] Ferrero M. C. Bregante J. Delpino M. V. Barrionuevo P. Fossati C. A. Giambartolomei G. H. (2011). Proinflammatory response of human endothelial cells to *Brucella* infection. *Microbes Infect.* 13 852–861. 10.1016/j.micinf.2011.04.010 21621633

[B63] Ferrero M. C. Fossati C. A. Baldi P. C. (2009). Smooth *Brucella* strains invade and replicate in human lung epithelial cells without inducing cell death. *Microbes Infect.* 11 476–483. 10.1016/j.micinf.2009.01.010 19397873

[B64] Ficapal A. Jordana J. Blasco J. M. Moriyón I. (1998). Diagnosis and epidemiology of *Brucella ovis* infection in rams. *Small Rumin. Res.* 29 13–19. 10.1016/S0921-4488(97)00108-9

[B65] Ficht T. A. Kahl-McDonagh M. M. Arenas-Gamboa A. M. Rice-Ficht A. C. (2009). Brucellosis: the case for live, attenuated vaccines. *Vaccine* 27 D40–D43. 10.1016/j.vaccine.2009.08.058 19837284 PMC2780424

[B66] Fontana C. Conde-Álvarez R. Ståhle J. Holst O. Iriarte M. Zhao Y. (2016). Structural studies of lipopolysaccharide-defective mutants from *Brucella melitensis* identify a core oligosaccharide critical in virulence. *J. Biol. Chem.* 291 7727–7741. 10.1074/jbc.M115.701540 26867577 PMC4817197

[B67] Forestier C. Deleuil F. Lapaque N. Moreno E. Gorvel J. P. (2000). *Brucella abortus* lipopolysaccharide in murine peritoneal macrophages acts as a down-regulator of T cell activation. *J. Immunol.* 165 5202–5210. 10.4049/jimmunol.165.9.5202 11046053

[B68] Forestier C. Moreno E. Pizarro-Cerdá J. Gorvel J. P. (1999). Lysosomal accumulation and recycling of lipopolysaccharide to the cell surface of murine macrophages, an *in vitro* and *in vivo* study. *J. Immunol.* 162 6784–6791. 10352299

[B69] Freer E. Moreno E. Moriyón I. Pizarro-Cerdá J. Weintraub A. Gorvel J. P. (1996). *Brucella-Salmonella* lipopolysaccharide chimeras are less permeable to hydrophobic probes and more sensitive to cationic peptides and EDTA than are their native *Brucella* sp. counterparts. *J. Bacteriol.* 178 5867–5876. 10.1128/jb.178.20.5867-5876.1996 8830680 PMC178440

[B70] Freer E. Pizarro-Cerdá J. Weintraub A. Bengoechea J. A. Moriyón I. Hultenby K. (1999). The outer membrane of *Brucella ovis* shows increased permeability to hydrophobic probes and is more susceptible to cationic peptides than are the outer membranes of mutant rough *Brucella abortus* strains. *Infect. Immun.* 67 6181–6186. 10.1128/IAI.67.11.6181-6186.1999 10531286 PMC97012

[B71] Galindo R. C. Muñoz P. M. de Miguel M. J. Marín C. M. Blasco J. M. Gortazar C. (2009). Differential expression of inflammatory and immune responses in rams experimentally infected with a rough virulent strain of *Brucella ovis*. *Vet. Immunol. Immunopathol.* 127 295–303. 10.1016/j.vetimm.2008.10.326 19056128

[B72] George L. Carmichael L. (1984). Antisperm responses in male dogs with chronic *Brucella canis* infections. *Am. J. Vet. Res.* 45 274–281. 6231870

[B73] Giambartolomei G. H. Scian R. Acosta-Rodríguez E. Fossati C. A. Delpino M. V. (2012). *Brucella abortus*-infected macrophages modulate T lymphocytes to promote osteoclastogenesis via IL-17. *Am. J. Pathol.* 181 887–896. 10.1016/j.ajpath.2012.05.029 22901753

[B74] Giambartolomei G. H. Zwerdling A. Cassataro J. Bruno L. Fossati C. A. Philipp M. T. (2004). Lipoproteins, not lipopolysaccharide, are the key mediators of the proinflammatory response elicited by heat-killed *Brucella abortus*. *J. Immunol.* 173 4635–4642. 10.4049/jimmunol.173.7.4635 15383598

[B75] Gil-Ramírez Y. Conde-Álvarez R. Palacios-Chaves L. Zúñiga-Ripa A. Grilló M. J. Arce-Gorvel V. (2014). The identification of wadB, a new glycosyltransferase gene, confirms the branched structure and the role in virulence of the lipopolysaccharide core of *Brucella abortus*. *Microb. Pathog.* 73 53–59. 10.1016/j.micpath.2014.06.002 24927935

[B76] Godfroid F. Cloeckaert A. Taminiau B. Danese I. Tibor A. de Bolle X. (2000). Genetic organization of the lipopolysaccharide O-antigen biosynthesis region of *Brucella melitensis* 16M (wbk). *Res. Microbiol.* 151 655–668. 10.1016/s0923-2508(00)90130-x 11081580

[B77] Godfroid F. Taminiau B. Danese I. Denoel P. A. Tibor A. Weynants V. E. (1998). Identification of the perosamine synthetase gene of *Brucella melitensis* 16M and involvement of lipopolysaccharide O side chain in *Brucella* survival in mice and in macrophages. *Infect. Immun.* 66 5485–5493. 10.1128/IAI.66.11.5485-5493.1998 9784561 PMC108687

[B78] Goldberg J. B. Pler G. B. (1996). *Pseudomonas aeruginosa* lipopolysaccharide and pathogenesis. *Trends Microbiol.* 4 490–494. 10.1016/s0966-842x(97)82911-3 9004407

[B79] Goldstein J. Hoffman T. Frasch C. Lizzio E. F. Beining P. R. Hochstein D. (1992). Lipopolysaccharide (LPS) from *Brucella abortus* is less toxic than that from *Escherichia coli*, suggesting the possible use of *B. abortus* or LPS from *B. abortus* as a carrier in vaccines. *Infect. Immun.* 60 1385–1389. 10.1128/IAI.60.4.1385-1389.1992 1548064 PMC257008

[B80] Grilló M. J. Blasco J. M. Gorvel J. P. Moriyón I. Moreno E. (2012). What have we learned from brucellosis in the mouse model? *Vet. Res.* 43:39. 10.1186/1297-9716-43-39 22500859 PMC3410789

[B81] Gul H. C. Erdem H. Bek S. (2009). Overview of neurobrucellosis.: a pooled analysis of 187 cases. *Int. J. Infect. Dis.* 13 e339–e343. 10.1016/j.ijid.2009.02.015 19428283

[B82] Hancock R. E. Mutharia L. M. Chan L. Darveau R. P. Speert D. P. Pier G. B. (1983). *Pseudomonas aeruginosa* isolates from patients with cystic fibrosis: a class of serum-sensitive, nontypable strains deficient in lipopolysaccharide O side chains. *Infect. Immun.* 42 170–177. 10.1128/iai.42.1.170-177.1983 6413410 PMC264539

[B83] Hensel M. E. Negron M. Arenas-Gamboa A. M. (2018). Brucellosis in dogs and public health risk. *Emerg. Infect. Dis.* 24 1401–1406. 10.3201/eid2408.171171 30014831 PMC6056133

[B84] High K. P. Prasad R. Marion C. R. Schurig G. G. Boyle S. M. Sriranganathan N. (2007). Outcome and immune responses after *Brucella abortus* infection in young adult and aged mice. *Biogerontology* 8 583–593. 10.1007/s10522-007-9106-6 17653832

[B85] Iriarte M. Gonzalez D. Delrue R. M. Monreal D. Conde R. Lopez-Goni I. (2004). “*Brucella* lipopolysaccharide: structure, biosynthesis and genetics,” in *Brucella: Molecular and Cellular Biology*, eds López-Goñi I. Moriyón I. (Pamplona: Horizon Bioscience), 159–191.

[B86] Jiménez de Bagüés M. P. Barberán M. Marín C. M. Blasco J. M. (1995). The *Brucella abortus* RB51 vaccine does not confer protection against *Brucella ovis* in rams. *Vaccine* 13 301–304. 10.1016/0264-410x(95)93317-3 7631517

[B87] Jiménez de Bagüés M. P. Elzer P. H. Jones S. M. Blasco J. M. Enright F. M. Schurig G. G. (1994). Vaccination with *Brucella abortus* rough mutant RB51 protects BALB/c mice against virulent strains of *Brucella abortus*, *Brucella melitensis*, and *Brucella ovis*. *Infect. Immun.* 62 4990–4996. 10.1128/IAI.62.11.4990-4996.1994 7927779 PMC303217

[B88] Jiménez de Bagüés M. P. Marín C. M. Barberán M. Blasco J. M. (1993). Evaluation of vaccines and of antigen therapy in a mouse model for *Brucella ovis*. *Vaccine* 11 61–66. 10.1016/0264-410x(93)90340-4 8427038

[B89] Jiménez de Bagüés M. P. Terraza A. Gross A. Dornand J. (2004). Different responses of macrophages to smooth and rough *Brucella* spp.: relationship to virulence. *Infect. Immun.* 72 2429–2433. 10.1128/iai.72.4.2429-2433.2004 15039375 PMC375206

[B90] Kahl-McDonagh M. M. Ficht T. A. (2006). Evaluation of protection afforded by *Brucella abortus* and *Brucella melitensis* unmarked deletion mutants exhibiting different rates of clearance in BALB/c mice. *Infect. Immun.* 74 4048–4057. 10.1128/IAI.01787-05 16790778 PMC1489724

[B91] Keleher L. L. Skyberg J. (2016). Activation of bovine neutrophils by *Brucella* spp. *Vet. Immunol. Immunopathol.* 177 1–6. 10.10106/j.vetimm.2016.05.011 27436438

[B92] Kerwin S. C. Lewis D. D. Hribernik T. N. Partington B. Hosgood G. Eilts B. E. (1992). Diskospondylitis associated with *Brucella canis* infection in dogs: 14 cases (1980-1991). *J. Am. Vet. Med. Assoc.* 201 1253–1257. 1429171

[B93] Kim S. Watarai M. Suzuki H. Makino S. Kodama T. Shirahata T. (2004). Lipid raft macrodomains mediate class A scavenger receptor-dependent infection of *Brucella abortus*. *Microb. Pathog.* 37 11–19. 10.1016/j.micpath.2004.04.002 15194155

[B94] Kubler-Kielb J. Vinogradov E. (2013). The study of the core part and non-repeating elements of the O-antigen of *Brucella* lipopolysaccharide. *Carbohydr. Res.* 366 33–37. 10.1016/j.carres.2012.11.004 23261780 PMC3540177

[B95] Lam J. S. Taylor V. L. Islam S. T. Hao Y. Hao Y. Kocincova D. (2011). Genetic and functional diversity of *Pseudomonas aeruginosa* lipopolysaccharide. *Front. Microbiol.* 2:118. 10.3389/fmicb.2011.00118 21687428 PMC3108286

[B96] Lapaque N. Moriyón I. Moreno E. Gorvel J. P. (2005). *Brucella* lipopolysaccharide acts as a virulence factor. *Curr. Opin. Microbiol.* 8 60–66. 10.1016/j.mib.2004.12.003 15694858

[B97] Li P. Tian M. Bao Y. Hu H. Liu J. Yin Y. (2017). *Brucella* rough mutant induce macrophage death via activating IRE1α pathway of endoplasmic reticulum stress by enhanced T4SS secretion. *Front. Cell. Infect. Microbiol.* 7:422. 10.3389/fcimb.2017.00422 29021973 PMC5623715

[B98] Liu Y. Sun J. Peng X. Dong H. Qin Y. Shen W. (2020). Deletion of the LuxR-type regulator VjbR in *Brucella canis* affects expression of type IV secretion system and bacterial virulence, and the mutant strain confers protection against *Brucella canis* challenge in mice. *Microb. Pathog.* 139:103865. 10.1016/j.micpath.2019.103865 31715318

[B99] Loutet S. A. Flannagan R. S. Kooi C. Sokol P. A. Valvano M. A. (2006). A complete lipopolysaccharide inner core oligosaccharide is required for resistance of *Burkholderia cenocepacia* to antimicrobial peptides and bacterial survival *in vivo*. *J. Bacteriol.* 188 2073–2080. 10.1128/JB.188.6.2073-2080.2006 16513737 PMC1428139

[B100] Macedo A. A. Silva A. P. Mol J. P. Costa L. F. Garcia L. N. Araújo M. S. (2015). The abcEDCBA-encoded ABC transporter and the virB operon-encoded type IV secretion system of *Brucella ovis* are critical for intracellular trafficking and survival in ovine monocyte-derived MPs. *PLoS One* 10:e0138131. 10.1371/journal.pone.0138131 26366863 PMC4569489

[B101] Maldonado R. F. Sá-Correia I. Valvano M. A. (2016). Lipopolysaccharide modification in gram-negative bacteria during chronic infection. *FEMS Microbiol. Rev.* 40 480–493. 10.1093/femsre/fuw007 27075488 PMC4931227

[B102] Mambres D. H. Machelart A. Potemberg G. De Trez C. Ryffel B. Letesson J. J. (2016). Identification of immune effectors essential to the control of primary and secondary intranasal infection with *Brucella melitensis* in mice. *J. Immunol.* 196 3780–3793. 10.4049/jimmunol.1502265 27036913

[B103] Mancilla M. (2015). Smooth to rough dissociation in *Brucella*: the missing link to virulence. *Front. Cell. Infect. Microbiol.* 5:98. 10.3389/fcimb.2015.00098 26779449 PMC4700419

[B104] Mancilla M. López-Goñi I. Moriyón I. Zárraga A. M. (2010). Genomic island 2 is an unstable genetic element contributing to *Brucella* lipopolysaccharide spontaneous smooth-to-rough dissociation. *J. Bacteriol.* 192 6346–6351. 10.1128/JB.00838-10 20952568 PMC3008527

[B105] Mancilla M. Marín C. M. Blasco J. M. Zárraga A. M. López-Goñi I. Moriyón I. (2012). Spontaneous excision of the O-polysaccharide wbkA glycosyltransferase gene is a cause of dissociation of smooth to rough *Brucella* colonies. *J. Bacteriol.* 194 1860–1867. 10.1128/JB.06561-11 22328663 PMC3318470

[B106] Manterola L. Guzmán-Verri C. Chaves-Olarte E. Barquero-Calvo E. de Miguel M. J. Moriyón I. (2007). BvrR/BvrS-controlled outer membrane proteins Omp3a and Omp3b are not essential for *Brucella abortus* virulence. *Infect. Immun.* 75 4867–4874. 10.1128/IAI.00439-07 17664262 PMC2044513

[B107] Martínez de Tejada G. Pizarro-Cerdá J. Moreno E. Moriyón I. (1995). The outer membranes of *Brucella* spp. are resistant to bactericidal cationic peptides. *Infect. Immun.* 63 3054–3061. 10.1128/IAI.63.8.3054-3061.1995 7622230 PMC173416

[B108] Martín-Martín A. I. Caro-Hernández P. Orduña A. Vizcaíno N. Fernández-Lago L. (2008). Importance of the Omp25/Omp31 family in the internalization and intracellular replication of virulent *B. ovis* in murine macrophages and HeLa cells. *Microbes Infect.* 10 706–710. 10.1016/j.micinf.2008.02.013 18457973

[B109] Martín-Martín A. I. Caro-Hernández P. Sancho P. Tejedor C. Cloeckaert A. Fernández-Lago L. (2009). Analysis of the occurrence and distribution of the Omp25/Omp31 family of surface proteins in the six classical *Brucella* species. *Vet. Microbiol.* 137 74–82. 10.1128/IAI.69.11.7020-7028.2001 19135812

[B110] Martín-Martín A. I. Sancho P. Tejedor C. Fernández-Lago L. Vizcaíno N. (2011). Differences in the outer membrane-related properties of the six classical *Brucella* species. *Vet. J.* 189 103–105. 10.1016/j.tvjl.2010.05.021 20576453

[B111] Martín-Martín A. I. Vizcaíno N. Fernández-Lago L. (2010). Cholesterol, ganglioside GM1, and class A scavenger receptor contribute to infection by *Brucella ovis* and *Brucella canis* in murine macrophages. *Microbes Infect.* 12 246–251. 10.1016/j.micinf.2009.12.008 20083220

[B112] Marzetti S. Carranza C. Roncallo M. Escobar G. I. Lucero N. E. (2013). Recent trends in human *Brucella canis* infection. *Comp. Immunol. Microbiol. Infect. Dis.* 36 55–61. 10.1016/j.cimid.2012.09.002 23040615

[B113] Meikle P. J. Perry M. B. Cherwonogrodzy J. W. Bundle D. R. (1989). Fine structure of A and M antigens from *Brucella* biovars. *Infect. Immun.* 57 2820–2828. 10.1128/iai.57.9.2820-2829.1989 2474504 PMC313533

[B114] Monreal D. Grilló M. J. González D. Marín C. M. de Miguel M. J. López-Goñi I. (2003). Characterization of *Brucella abortus* O-polysaccharide and core lipopolysaccharide mutants and demonstration that a complete core is required for rough vaccines to be efficient against *Brucella abortus* and *Brucella ovis* in the mouse model. *Infect. Immun.* 71 3261–3271. 10.1128/iai.71.6.3261-3271.2003 12761107 PMC155776

[B115] Moore J. A. Kakuk T. J. (1969). Male dogs naturally infected with *Brucella canis*. *J. Am. Vet. Med. Assoc.* 155 1352–1358. 4936877

[B116] Moran M. C. Bence A. R. Vallecillo M. F. S. Lützelschwab C. M. Rodriguez M. G. Pardo R. (2021). Polymeric antigen BLSOmp31 formulated with class B CpG-ODN in a nanostructure (BLSOmp31/CpG-ODN/Coa-ASC16) administered by parenteral or mucosal routes confers protection against *Brucella ovis* in Balb/c mice. *Res. Vet. Sci.* 135 217–227. 10.1016/j.rvsc.2021.02.011 33631456

[B117] Moreno E. (2014). Retrospective and prospective perspectives on zoonotic brucellosis. *Front. Microbiol.* 13:213. 10.3389/fmicb.2014.00213 24860561 PMC4026726

[B118] Moreno E. Berman D. T. Boettcher L. A. (1981). Biological activities of *Brucella abortus* lipopolysaccharides. *Infect. Immun.* 31 362–370. 10.1128/IAI.31.1.362-370.1981 6783538 PMC351792

[B119] Moreno E. Jones L. M. Berman D. T. (1984). Immunochemical characterization of rough *Brucella* lipopolysaccharides. *Infect. Immun.* 43 779–782. 10.1128/IAI.43.3.779-782.1984 6421737 PMC264247

[B120] Moriyón I. Grilló M. J. Monreal D. González D. Marín C. López-Goñi I. (2004). Rough vaccines in animal brucellosis: structural and genetic basis and present status. *Vet. Res.* 35 1–38. 10.1051/vetres:2003037 15099501

[B121] Murphy E. A. Sathiyaseelan J. Parent M. A. Zou B. Baldwin C. L. (2001). Interferon-γ is crucial for surviving a *Brucella abortus* infection in both resistant C57BL/6 and susceptible BALB/c mice. *Immunology* 103 511–518. 10.1046/j.1365-2567.2001.01258.x 11529943 PMC1783270

[B122] Palmer M. V. Cheville N. F. (1997). Effects of oral or intravenous inoculation with *Brucella abortus* strain RB51 vaccine in beagles. *Am. J. Vet. Res.* 58 851–856. 9256969

[B123] Palomares-Resendiz E. Arellano-Reynoso B. Hernández-Castro R. Tenorio-Gutiérrez V. Salas-Téllez E. Súarez-Güemes F. (2012). Immunogenic response of *Brucella canis* virB10 and virB11 mutants in a murine model. *Front. Cell. Infect. Microbiol.* 2:25. 10.3389/fcimb.2012.00035 22919627 PMC3417389

[B124] Paolicchi F. A. Casaro P. A. Gimenoc E. J. Kortebanid L. G. Mazzolli A. B. (2000). Antisperm responses in rams experimentally infected with *B. ovis*. *Small Rum. Res.* 36 7–15. 10.1016/s0921-4488(99)00108-x

[B125] Pappas G. Panagopoulou P. Christou L. Akritidis N. (2008). Biological weapons. *Cell. Mol. Life Sci.* 63 2229–2236. 10.1007/s00018-006-6311-4 16964579 PMC11136069

[B126] Pappas G. Papadimitriou P. Akritidis N. Christou L. Tsianos E. V. (2006). The new global map of human brucellosis. *Lancet Infect. Dis.* 6 91–99. 10.1016/S1473-3099(06)70382-6 16439329

[B127] Pascual D. W. Yang X. Wang H. Goodwin Z. Hoffman C. Clapp B. (2019). Alternative strategies for vaccination to brucellosis. *Microbes Infect.* 20 599–605. 10.1016/j.micinf.2017.12.006 29287984 PMC6019614

[B128] Pei J. Turse J. E. Ficht T. A. (2008). Evidence of *Brucella abortus* OPS dictating uptake and restricting NF-kappaB activation in murine macrophages. *Microbes Infect.* 10 582–590. 10.1016/j.micinf.2008.01.005 18457975 PMC2752336

[B129] Pei J. Turse J. E. Wu Q. Ficht T. A. (2006). *Brucella abortus* rough mutants induce macrophage oncosis that requires bacterial protein synthesis and direct interaction with the macrophage. *Infect. Immun.* 74 2667–2675. 10.1128/IAI.74.5.2667-2675.2006 16622203 PMC1459739

[B130] Pérez-Etayo L. de Miguel M. J. Conde-Álvarez R. Muñoz P. M. Khames M. Iriarte M. (2018). The CO2-dependence of *Brucella ovis* and *Brucella abortus* biovars is caused by defective carbonic anhydrases. *Vet. Res.* 49:85. 10.1186/s13567-018-0583-1 30185220 PMC6126018

[B131] Perkins S. D. Smither S. J. Atkins H. S. (2010). Towards a *Brucella* vaccine for humans. *FEMS Microbiol. Rev.* 34 379–394. 10.1111/j.1574-6976.2010.00211.x 20180858

[B132] Piampiano P. McLeary M. Young L. W. Janner D. (2000). Brucellosis: unusual presentations in two adolescent boys. *Pediatr. Radiol.* 30 355–357. 10.1007/s002470050760 10836605

[B133] Porte F. Naroeni A. Ouahrani-Bettache S. Liautard J. P. (2003). Role of the *Brucella suis* lipopolysaccharide O antigen in phagosomal genesis and in inhibition of phagosome-lysosome fusion in murine macrophages. *Infect. Immun.* 71 1481–1490. 10.1128/iai.71.3.1481-1490.2003 12595466 PMC148865

[B134] Pujol C. Klein K. A. Romanov G. A. Palmer L. E. Cirota C. Zhao Z. (2009). Yersinia pestis can reside in autophagosomes and avoid xenophagy in murine macrophages by preventing vacuole acidification. *Infect. Immun.* 77 2251–2261. 10.1128/IAI.00068-09 19289509 PMC2687347

[B135] Pujol M. Borie C. Montoya M. Ferreria A. Vernal R. (2019). *Brucella canis* induces CD4+ T cells multi-cytokine Th1/Th17 production via dendritic cell activation. *Comp. Immunol. Microbiol. Infect. Dis.* 62 68–75. 10.1016/j.cimid.2018.11.017 30711049

[B136] Pujol M. Castillo F. Alvarez C. Rojas C. Borie C. Ferreira A. (2017). Variability in the response of canine and human dendritic cells stimulated with *Brucella canis*. *Vet. Res.* 48:72. 10.1186/s13567-017-0476-8 29096717 PMC5667440

[B137] Qian J. Bu Z. Lang X. Yan G. Yang Y. Wang X. (2017). A safe and molecular-tagged *Brucella canis* ghosts confers protection against virulent challenge in mice. *Vet. Microbiol.* 204 121–128. 10.1016/j.vetmic.2017.04.027 28532790

[B138] Rajashekara G. Covert J. Petersen E. Eskra L. Splitter G. (2008). Genomic island 2 of *Brucella melitensis* is a major virulence determinant: functional analyses of genomic islands. *J. Bacteriol.* 190 6243–6252. 10.1128/JB.00520-08 18641138 PMC2546784

[B139] Ramamoorthy S. Woldemeskel M. Ligett A. Snider R. Cobb R. Rajeev S. (2011). *Brucella suis* infection in dogs, Georgia, USA. *Emerg. Infect. Dis.* 17 2386–2387. 10.3201/eid171.111127 22172146 PMC3311166

[B140] Reeves E. P. Ali T. Leonard P. Hearty S. O’Kennedy R. May F. E. (2008). *Helicobacter pylori* lipopolysaccharide interacts with TFF1 in a pH-dependent manner. *Gastroenterology* 135 2043–2054. 10.1053/j.gastro.2008.08.049 18848942

[B141] Reeves P. (1995). Role of O-antigen variation in the immune response. *Trends Microbiol.* 3 381–386. 10.1016/s0966-842x(00)88983-0 8564356

[B142] Ridler A. L. West D. M. (2011). Control of *Brucella ovis* infection in sheep. *Vet. Clin. North Am. Food Anim. Pract.* 27 61–66. 10.1016/j.cvfa.2010.10.013 21215890

[B143] Riley L. K. Robertson D. C. (1984). Brucellacidal activity of human and bovine polymorphonuclear leukocyte granule extracts against smooth and rough strains of *Brucella abortus*. *Infect. Immun.* 46 231–236. 10.1128/IAI.46.1.231-236.1984 6090316 PMC261460

[B144] Rittig M. G. Kaufmann A. Robins A. Shaw B. Sprenger H. Gemsa D. (2003). Smooth and rough lipopolysaccharide phenotypes of *Brucella* induce different intracellular trafficking and cytokine/chemokine release in human monocytes. *J. Leukoc. Biol.* 74 1045–1055. 10.1189/jlb.0103015 12960272

[B145] Roop R. M. II Barton I. S. Hopersberger D. Martin D. W. (2021). Uncovering the hidden credentials of *Brucella* virulence. *Microbiol. Mol. Biol. Rev.* 85:e00021-19. 10.1128/MMBR.00021-19 33568459 PMC8549849

[B146] Sá J. C. Silva T. M. Costa E. A. Silva A. P. Tsolis R. M. Paixão T. A. (2012). The virB-encoded type IV secretion system is critical for establishment of infection and persistence of *Brucella ovis* infection in mice. *Vet. Microbiol.* 159 130–140. 10.1016/j.vetmic.2012.03.029 22483850

[B147] Saldías M. S. Ortega X. Valvano M. A. (2009). *Burkholderia cenocepacia* O antigen 953 lipopolysaccharide prevents phagocytosis by macrophages and adhesion to epithelial 954 cells. *J. Med. Microbiol.* 58 1542–1548. 10.1099/jmm.0.013235-0 19713359

[B148] Salvador-Bescós M. Gil-Ramírez Y. Zúñiga-Ripa A. Martínez-Gómez E. de Miguel M. J. Muñiz P. M. (2018). WadD, a new *Brucella* lipopolysaccharide core glycosyltransferase identified by genomic search and phenotypic characterization. *Front. Microbiol.* 9:2292. 10.3389/fmicb.2018.02293 30319590 PMC6171495

[B149] Sancho P. Tejedor C. Sidhu-Muñoz R. S. Fernández-Lago L. Vizcaíno N. (2014). Evaluation in mice of *Brucella ovis* attenuated mutants for use as live vaccines against *B. ovis* infection. *Vet. Res.* 45:61. 10.1186/1297-9716-45-61 24898325 PMC4057616

[B150] Schurig G. G. Roop R. M. II Bagchi T. Boyle S. Buhrman D. Sriranganathan N. (1991). Biological properties of RB51; a stable rough strain of *Brucella abortus*. *Vet. Microbiol.* 2 171–188. 10.1016/0378-1135(91)90091-s 1908158

[B151] Schwab U. Abdullah L. H. Perlmutt O. S. Albert D. Davis C. W. Arnold R. R. (2014). Localization of *Burkholderia cepacia* 963 complex bacteria in cystic fibrosis lungs and interactions with *Pseudomonas aeruginosa* 964 in hypoxic mucus. *Infect. Immun.* 82 4729–4745. 10.1128/iai.01876-14 25156735 PMC4249344

[B152] Shimoya K. Moriyama A. Matsuzaki N. Ogata I. Koyama M. Azuma C. (1999). Human placental cells show enhanced production of interleukin (IL)-8 in response to lipopolysaccharide (LPS), IL-1, and tumour necrosis factor (TNF)-alpha, but not IL-6. *Mol. Hum. Reprod.* 5:885. 10.1093/molehr.5.9.885 10460229

[B153] Silva A. P. Macêdo A. A. Silva T. M. Ximenes L. C. Brandão H. M. Paixão T. A. (2015). Protection provided by an encapsulated live attenuated ΔabcBA strain of *Brucella ovis* against experimental challenge in a murine model. *Clin. Vaccine Immunol.* 22 789–797. 10.1128/CVI.00191-15 25947146 PMC4478529

[B154] Silva T. M. Macêdo A. A. Costa L. F. Rocha C. E. Garcia L. N. Farias J. R. (2015). Encapsulated *Brucella ovis* lacking a putative ATP-binding cassette transporter (ΔabcBA) protects against wild type *Brucella ovis* in rams. *PLoS One* 10:e0136865. 10.1371/journal.pone.0136865 26317399 PMC4552948

[B155] Silva T. M. Mol J. P. Winter W. G. Atluri V. Xavier M. N. Pires S. F. (2014). The predicted ABC transporter AbcEDCBA is required for type IV secretion system expression and lysosomal evasion by *Brucella ovis*. *PLoS One* 9:e114532. 10.1371/journal.pone.0114532 25474545 PMC4256435

[B156] Silva T. M. Paixão T. A. Costa E. A. Xavier M. N. Sá J. C. Moustacas V. S. (2011). Putative ATP-binding cassette transporter is essential for *Brucella ovis* pathogenesis in mice. *Infect. Immun.* 79 1706–1717. 10.1128/IAI.01109-10 21300772 PMC3067543

[B157] Singh C. Hwayoung L. Tian Y. Barta S. S. However S. Fujimoto L. M. (2020). Mutually constructive roles of Ail and LPS in *Yersinia pestis* serum survival. *Mol. Microbiol.* 114 510–520. 10.1111/mmi.14530 32462782 PMC7594906

[B158] Skendros P. Boura P. (2013). Immunity to brucellosis. *Rev. Sci. Tech.* 32 137–147. 10.20506/rst.32.1.2190 23837372

[B159] Smith J. A. (2018). *Brucella* lipopolysaccharide and pathogenicity: the core of the matter. *Virulence* 9 379–382. 10.1080/21505594.2017.1395544 29144201 PMC7000210

[B160] Soler-Lloréns P. Gil-Ramírez Y. Zabalza-Baranguá A. Iriarte M. Conde-Álvarez R. Zúñiga-Ripa A. (2014). Mutants in the lipopolysaccharide of *Brucella ovis* are attenuated and protect against *B. ovis* infection in mice. *Vet. Res.* 45:72. 10.1186/s13567-014-0072-0 25029920 PMC4107470

[B161] Starr T. Ng T. W. Wehrly T. D. Knodler L. A. Celli J. (2008). *Brucella* intracellular replication requires trafficking through the late endosomal/lysosomal compartment. *Traffic* 9 678–694. 10.1111/j.1600-0854.2009.00718.x 18266913

[B162] Stranahan L. W. Chaki S. P. Garcia-Gonzalez D. G. Khalaf O. K. Arenas-Gamboa A. M. (2020). Evaluation of the efficacy of *Brucella canis* RM6/66 ΔvjbR vaccine candidate for protection against *B. canis* infection in mice. *mSphere* 5:e00172-20. 10.1128/mSphere.00172-20 32434839 PMC7380573

[B163] Stranahan L. W. Khalaf O. H. Garcia-Gonzalez D. G. Arenas-Gamboa A. M. (2019). Characterization of *Brucella canis* infection in mice. *PLoS One* 14:e0218809. 10.1371/journal.pone.0218809 31220185 PMC6586350

[B164] Suárez-Esquivel M. Ruiz-Villalobos N. Hidalgo-Jara W. Chacón-Díaz C. Zúñiga-Pereira A. M. Masís-Mora M. (2021). Canine brucellosis in Costa Rica reveals widespread *Brucella canis* infection and the recent introduction of foreign strains. *Vet. Microbiol.* 257:109072. 10.1016/j.vetmic.2021.109072 33965789

[B165] Surendran N. Hiltbold E. M. Heid B. Akira S. Standiford T. J. Sriranganathan N. (2012). Role of TLRs in *Brucella* mediated murine DC activation in vitro and clearance of pulmonary infection *in vivo*. *Vaccine* 30 1502–1512. 10.1016/j.vaccine.2011.12.036 22234268

[B166] Szabo I. Grafe M. Kemper N. Junker E. Malorny B. (2017). Genetic basis for loss of immunoreactive O-chain in *Salmonella enterica* serovar Enteritidis veterinary isolates. *Vet. Microbiol.* 204 165–173. 10.1016/j.vetmic.2017.03.033 28532797

[B167] Tian M. Qu J. Han X. Ding C. Wang S. Peng D. (2014). Mechanism of Asp24 upregulation in *Brucella abortus* rough mutant with a disrupted O-antigen export system and effect of Asp24 in bacterial intracellular survival. *Infect. Immun.* 82 2840–2850. 10.1128/IAI.01765-14 24752516 PMC4097617

[B168] Truong Q. L. Cho Y. Kim K. Park B. K. Hahn T. W. (2015). Booster vaccination with safe, modified, live-attenuated mutants of *Brucella abortus* strain RB51 vaccine confers protective immunity against virulent strains of *B. abortus* and *Brucella canis* in BALB/c mice. *Microbiology* 161 2137–2148. 10.1099/mic.0.000170 26341622

[B169] Tsolis R. M. Seshadri R. Santos R. L. Sangari F. J. Lobo J. M. de Jong M. F. (2009). Genome degradation in *Brucella ovis* corresponds with narrowing of its host range and tissue tropism. *PLoS One* 4:e5519. 10.1371/journal.pone.0005519 19436743 PMC2677664

[B170] Tumurkhuu G. Koide N. Takahashi K. Hassan F. Islam S. Ito H. (2006). Characterization of biological activities of *Brucella melitensis* lipopolysaccharide. *Microbiol. Immunol.* 50 421–427. 10.1111/j.1348-0421.2006.tb03810.x 16785713

[B171] Turse J. E. Pei J. Ficht T. A. (2011). Lipopolysaccharide-deficient *Brucella* variants arise spontaneously during infection. *Front. Microbiol.* 23:54. 10.3389/fmicb.2011.00054 21833310 PMC3153030

[B172] Ugalde J. E. Czibener C. Feldman M. F. Ugalde R. A. (2000). Identification and characterization of the *Brucella abortus* phosphoglucomutase gene: role of lipopolysaccharide in virulence and intracellular multiplication. *Infect. Immun.* 68 5716–5723. 10.1128/iai.68.10.5716-5723.2000 10992476 PMC101528

[B173] Usta M. Aras Z. Tas A. (2012). Oxidant and antioxidant parameters in patients with *Brucella canis*. *Clin. Biochem.* 45 366–367. 10.1016/j.clinbiochem.2011.12.028 22266398

[B174] Vitry M. A. De Trez C. Goriely S. Dumoutier L. Akira S. Ryffel B. (2012). Crucial role of gamma interferon-producing CD4+ Th1 cells but dispensable function of CD8+ T cell, B cell, Th2, and Th17 responses in the control of *Brucella melitensis* infection in mice. *Infect. Immun.* 80 4271–4280. 10.1128/IAI.00761-12 23006848 PMC3497404

[B175] Vizcaíno N. Caro-Hernández P. Cloeckaert A. Fernández-Lago L. (2004). DNA polymorphism in the omp25/omp31 family of *Brucella* spp. identification of a 1.7-kb inversion in *Brucella cetaceae* and of a 15.1-kb genomic island, absent from *Brucella ovis*, related to the synthesis of smooth lipopolysaccharide. *Microbes Infect.* 6 821–824. 10.1016/j.micinf.2004.04.009 15374004

[B176] Wang H. Hoffman C. Yang X. Clapp B. Pascual D. W. (2020). Targeting resident memory T cell immunity culminates in pulmonary and systemic protection against *Brucella* infection. *PLoS Pathog.* 16:e1008176. 10.1371/journal.ppat.1008176 31951645 PMC6968852

[B177] Wang L. Cui J. Misner M. B. Zhang Y. (2018). Sequencing and phylogenetic characterization of *Brucella canis* isolates, Ohio, 2016. *Transbound. Emerg. Dis.* 65 944–948. 10.1111/tbed.12902 29752779

[B178] Wang L. Wang Q. Reeves P. R. (2010). The variation of O antigens in gram-negative bacteria. *Subcell. Biochem.* 53 123–152. 10.1007/978-90-481-9078-2_6 20593265

[B179] Wattam A. R. Williams K. P. Snyder E. E. Almeida N. F. Jr. Shukla M. Dickerman A. W. (2009). Analysis of ten *Brucella* genomes reveals evidence for horizontal gene transfer despite a preferred intracellular lifestyle. *J. Bacteriol.* 191 3569–3579. 10.1128/JB.01767-08 19346311 PMC2681906

[B180] Whatmore A. M. (2009). Current understanding of the genetic diversity of *Brucella*, an expanding genus of zoonotic pathogens. *Infect. Genet. Evol.* 9 1168–1184. 10.1016/j.meegid.2009.07.001 19628055

[B181] Winter A. J. Schurig G. Boyle S. M. Sriranganathan N. Bevins J. S. Enrigh F. M. (1996). Protection of BALB/c mice against homologous and heterologous species of *Brucella* by rough strain vaccines derived from *Brucella melitensis* and *Brucella suis* biovar 4. *Am. J. Vet. Res.* 57 677–683. 8723881

[B182] Yang K. He Y. Park C. G. Kang Y. S. Zhang P. Han Y. (2019). *Yersinia pestis* interacts with SIGNR1 (CD209b) for promoting host dissemination and infection. *Front. Immunol.* 10:96. 10.3389/fimmu.2019.00096 30915064 PMC6422942

[B183] Zavattieri L. Ferrero M. C. Alonso Paiva I. M. Sotelo A. D. Canellada A. M. Baldi P. C. (2020). *Brucella abortus* proliferates in decidualized and non-decidualized human endometrial cells inducing a proinflammatory response. *Pathogens* 9:369. 10.3390/pathogens9050369 32408491 PMC7281465

[B184] Zwerdling A. Delpino M. V. Barrionuevo P. Cassataro J. Pasquevich K. A. García Samartino C. (2008). *Brucella* lipoproteins mimic dendritic cell maturation induced by *Brucella abortus*. *Microbes Infect.* 10 1346–1354. 10.1016/j.micinf.2008.07.035 18761420

[B185] Zygmunt M. S. Blasco J. M. Letesson J. J. Cloeckaert J. Moriyón I. (2009). DNA polymorphism analysis of *Brucella* lipopolysaccharide genes reveals marked differences in O-polysaccharide biosynthetic genes between smooth and rough *Brucella* species and novel species-specific markers. *BMC Microbiol.* 159:130–140. 10.1016/j.vetmic.2012.03.029 19439075 PMC2698832

[B186] Zygmunt M. S. Jacques I. Bernardet N. Cloeckaert A. (2012). Lipopolysaccharide heterogeneity in the atypical group of novel emerging *Brucella* species. *Clin. Vaccine Immunol.* 19 1370–1373. 10.1128/CVI.00300-12 22761298 PMC3428386

